# ALVAC-prime and monomeric gp120 protein boost induces distinct HIV-1 specific humoral and cellular responses compared with adenovirus-prime and trimeric gp140 protein boost

**DOI:** 10.1371/journal.pgph.0004250

**Published:** 2025-04-11

**Authors:** Leigh H. Fisher, Erica Lazarus, Chenchen Yu, Zoe Moodie, Daniel J. Stieh, Nicole Yates, Lu Zhang, Sheetal Sawant, Stephen C. De Rosa, Kristen W. Cohen, Daryl Morris, Shannon Grant, April Randhawa, Maurine D. Miner, Jenny Hendriks, Frank Wegmann, Katherine M. Gill, Fatima Laher, Linda-Gail Bekker, Glenda E. Gray, Lawrence Corey, M. Juliana McElrath, Troy Martin, Peter B. Gilbert, Georgia Tomaras, Stephen R. Walsh, Lindsey R. Baden, The HVTN 100, HVTN 117/HPX2004 study teams

**Affiliations:** 1 Vaccine and Infectious Disease Division, Fred Hutchinson Cancer Center, Seattle, Washington, United States of America; 2 Perinatal HIV Research Unit, University of the Witwatersrand, Johannesburg, South Africa; 3 Janssen Vaccines & Prevention, Leiden, The Netherlands; 4 Duke Human Vaccine Institute, Duke University, Durham, North Carolina, United States of America; 5 Duke University, Durham, North Carolina, United States of America; 6 The Desmond Tutu HIV Centre, University of Cape Town, Cape Town, South Africa; 7 Perinatal HIV Research Unit, Faculty of Health Sciences, University of the Witwatersrand, Soweto, South Africa; 8 South African Medical Research Council, Cape Town, South Africa; 9 Department of Biostatistics, University of Washington, Seattle, Washington, United States of America; 10 Department of Surgery, Duke University Medical Center, Durham, North Carolina, United States of America; 11 Duke Human Vaccine Institute, Duke University Medical Center, Durham, North Carolina, United States of America; 12 Department of Immunology, Duke University Medical Center, Durham, North Carolina, United States of America; 13 Department of Molecular Genetics and Microbiology, Duke University Medical Center, Durham, North Carolina, United States of America; 14 Division of Infectious Diseases, Brigham and Women’s Hospital, Boston, Massachusetts, United States of America; 15 Harvard Medical School, Boston, Massachusetts, United States of America; 16 Center for Virology and Vaccine Research, Beth Israel Deaconess Medical Center, Boston, Massachusetts, United States of America; Universidade Catolica Portuguesa, PORTUGAL

## Abstract

Although clade-specific and cross-clade mosaic prime–boost HIV-1 vaccine regimens were advanced to the HVTN 702 and HVTN 705 efficacy trials, neither regimen prevented HIV acquisition. The respective Phase 1/2a studies, HVTN 100 (NCT02404311) and HVTN 117/HPX2004 (NCT02788045), provided rich immunological data, including previously identified correlates of risk, for comparing immune responses elicited by these vaccine regimens over time. We analyzed antibody responses measured by binding antibody multiplex assay, and CD4+ and CD8+ T-cell responses measured by intracellular cytokine staining in per-protocol vaccinees in HVTN 100 (n=186) vs. HVTN 117/HPX2004 (n=99) after the months 6 and 12 vaccinations (months 6.5/7 and 12.5/13), and 6 months after the last vaccination (month 18). At month 12.5/13, both regimens induced similarly high IgG breadth against gp120, gp140, and V1V2 antigens, and similar IgG responses to gp70-BCaseA V1V2. IgG V1V2 responses were more durable in HVTN 117/HPX2004, with the largest difference in the gp70-BCaseA V1V2 IgG response rate at month 18 (17.8% in HVTN 100 vs 61.9% in HVTN 117/HPX2004, p<0.001). IgG3 responses to consensus Env antigens were higher and more durable in HVTN117/HPX2004; for example, IgG3 response rate to the consensus gp140 antigen was 65.9% in HVTN 117/HPX2004 vs 6.3% in HVTN 100 at month 18 (TMLE p<0.0001). At month 18, both regimens induced similar IgG3 responses to gp70-BCaseA V1V2 (3.2% in HVTN 100 vs 1.1% in HVTN 117/HPX2004). Polyfunctional CD4+ Env was significantly higher in HVTN 100, and polyfunctional CD4+ Gag was higher in HVTN 117/HPX2004. CD8+ T-cell responses were not seen in HVTN 100, while CD8+ T-cell response rates in HVTN 117/HPX2004 reached up to 42%. Despite the distinct immune responses induced by the two HIV vaccine regimens, the lack of demonstrated efficacy suggests that broader, higher magnitude, and possibly qualitatively different immune responses are needed for protection against HIV acquisition.

**Trial registration:** ClinicalTrials.gov NCT02404311 and NCT02788045; South African National Clinical Trials Registry (DOH-27-0215-4796)

## 1 Introduction

Development of a preventive HIV-1 vaccine remains a pressing global health priority [1]. Six vaccine concepts have completed evaluation in clinical trials [2–6]. To date only the RV144 clade B/E canarypox viral vector (ALVAC-HIV (vCP1521)) prime boosted with AIDSVAX clade B/E envelope (Env) glycoprotein 120 (gp120) has shown moderate protection against HIV-1 acquisition [5]. A case-control analysis of RV144 identified antibodies to a scaffolded region of the conserved V1V2 loop to correlate inversely with infection while serum IgA antibodies binding to Env correlated directly with infection. However, no increase in HIV acquisition compared to placebo was seen in vaccine recipients with high levels of vaccine-induced Env-specific plasma IgA antibodies [7]. Antibody dependent cellular cytotoxicity (ADCC), avidity, and CD4+ T-cell responses to Env also correlated with reduced acquisition [8]. These findings suggest that RV144 protective antibody responses were mediated through V1V2 directed IgG3 (and not IgG2 or IgG4), and the mechanism of protection is through antibody Fc directed ADCC of V1V2 displaying targets in the setting of low levels of Env IgA, which compete for the same region.

Building upon the modest success of the RV144 vaccine regimen, multiple trials of immunogenicity and safety were conducted in South Africa: one of them assessed the identical RV144 regimen (HVTN 097, phase 1b) [9]; and HVTN 100 (phase 1/2), another ALVAC/protein regimen modified for clade C, the predominant strain of HIV-1 in southern Africa [10]. The HVTN 100 regimen differed from that of RV144 in several ways: both the ALVAC and Env gp120 vaccines had been modified to express clade C immunogens; the alum-based adjuvant was replaced with MF59 in an effort to improve immunogenicity; and a month 12 booster was added to increase durability. While HVTN 097 did not move forward to efficacy testing, qualifying immunogenicity results from the HVTN 100 trial triggered the HVTN 702 efficacy trial (NCT02968849) in South Africa. Following interim Data Safety and Monitoring Board (DSMB) review, the HVTN 702 trial showed that while the vaccines were safe, they did not reduce HIV acquisition [11].

A contrasting approach to geographically matching immunogens to locally circulating strains of HIV-1 is to use computationally-derived mosaic antigens for the development of a cross-clade globally-applicable vaccine regimen [12]. Mosaic antigens are in silico-derived, full-length recombinant proteins designed to maximize potential coverage of T-cell epitopes and linear B-cell epitopes, and to allow natural expression, antigen processing, and presentation. Studies of mosaic HIV-1 antigens in rhesus macaques have shown that these mosaic sequences increase the breadth and depth of T-cell immune responses compared with either consensus or natural sequences [13,14]. Mosaic HIV-1 antigens have been tested in clinical trials using both poxvirus vectors [15] and adenovirus serotype 26 (Ad26) vectors [16].

In the multinational APPROACH study, a trivalent Ad26 mosaic vector (Ad26.Mos.HIV) boosted with an alum-adjuvanted trimeric clade C gp140 Env protein was found to elicit a more favorable immunogenicity profile than comparator regimens in approximately 400 participants in southern Africa, Thailand, and the United States (NCT02315703) [16]. To improve coverage of the most common global strain, clade C, a tetravalent set of mosaic inserts (Ad26.Mos4.HIV) was developed and tested in Rwanda and the United States in the TRAVERSE HVTN 117/HPX2004 trial (NCT02788045) [17]. The potent cross-clade immunogenicity seen in the Ad26.Mos4.HIV (Group 2A) arm [18] triggered the launch of the HVTN 705/HPX2008 proof-of-concept efficacy trial (NCT03060629) in young women in southern Africa. A complementary efficacy trial opened in October 2019, using the same tetravalent Ad26 mosaic priming vaccine but boosted with a combined clade C and mosaic trimeric gp140 Env [HVTN 706/HPX3002; NCT03964415] in the Americas and Europe in a population of men and transgender individuals who have sex with men. Both HVTN 705/HPX2008 & 706/HPX3002 were completed early due to lack of efficacy at preventing HIV-1 acquisition [19,20].

Pre-clinical investigations into both the HVTN 702 and HVTN 705/HPX2008 regimens provided insights into potential immune markers of interest. Kaur and Vaccari (2024) [21] summarized the history of nonhuman primate (NHP) studies of ALVAC-vector based and Ad26-vector based HIV/SIV/SHIV vaccines that have been conducted. NHP challenge studies of the HVTN 705/HPX2008 vaccine regimen identified Env-specific IgG binding antibodies, antibody-dependent phagocytosis markers, T-cell responses measured by ELISpot, and V1V2-specific IgG binding antibodies as immune correlates of SHIV acquisition [13,22]. An NHP challenge study of the HVTN 100 vaccine regimen, and of a similar regimen with the gp120 delivered with alum, showed efficacy in delaying the onset of SIVmac251 for the alum but not the MF59 adjuvant, and found that mucosal IgG V2 antibodies correlated with the observed efficacy [23].

Recent correlates of risk analyses from both the HVTN 702 and HVTN 705/HPX2008 efficacy trials [24,25] suggested that higher levels of immune responses may be necessary to provide protection. Specifically, the HVTN 702 immunogenicity and immune correlates analysis found IgG binding antibodies to the V1V2 region of Env responses were much higher in RV144 compared to the responses in HVTN 702 [11]. However, the HVTN 702 and HVTN 705/HPX2008 correlates analyses were limited in the sample size and immunological endpoints compared to the available data from the earlier phase trials. For example, compared to the limited case-control study in HVTN 702, HVTN 100 assessed more immunological endpoints from more study participants. Moreover, both early phase studies provided immunological responses from multiple time points, including 6 months after the last vaccination.

Building on the HVTN 702 correlates analysis, which included comparisons to RV144, HVTN 097, and HVTN 100, the goal of this study was to compare responses from HVTN 100 and HVTN 117/HPX2004 to better understand the similarities and differences in the immune responses elicited by these two vaccine regimens. We leveraged the existing immunological data from each of the early phase trials from multiple time points. As no further specimens were run, we included all the available data that was comparable between the two studies. We focused on the leading immune correlates identified in the three efficacy trials (e.g., IgG and IgG3 binding antibodies against Env V1V2 and polyfunctional CD4+ T cells against Env peptides), but also included additional endpoints with available data (e.g., CD8+ T-cell responses) [7,26,27]. The current study included markers derived from immunoassays that were shown to be correlates of protection in NHP models and for which there were data available from HVTN 100 and HVTN 705/HPX2008, which amounted to Env- and V1V2-specific IgG binding antibodies. A positive control with observed partial efficacy and immune correlates, such as RV144, was not included in this study because comparable data from RV144 were not available for many of the immune response markers presented, and, where available, the comparisons with HVTN 100 have already been published [10,11,24].

## 2 Materials and methods

### 2.1 Included protocols

This cross-protocol analysis compared immune responses from participants in HVTN 100 Group 1 and HVTN 117/HPX2004 Subgroup 2A, who received the vaccine regimens that were subsequently investigated in HVTN 702 and HVTN 705/HPX2008, respectively. Both trials were randomized, double-blind, placebo-controlled, phase 1/2 studies assessing preventive HIV vaccine regimens. HVTN 100 enrolled participants between Feb 9, 2015 and May 26, 2015; HVTN 117/HPX2004 enrolled participants between July 12, 2016 and Aug 27, 2018. These trials have each been described in detail elsewhere [10,17]. The data for this analysis was accessed on August 5, 2019 for research purposes and the authors did not have access to information that could identify individual participants.

### 2.2 Participants

Volunteers were eligible if they were adults in overall good health, living without HIV-1, able to give written informed consent, had a low likelihood for HIV acquisition per local guidelines, and had not previously received an HIV vaccine. For HVTN 100, eligible volunteers were aged 18-40 years, and 18-50 years for HVTN 117/HPX2004. In both studies, female participants were required to be on contraception, not pregnant, and non-lactating; in addition, for HVTN 117/HPX2004 only, female partners of male participants were required to use contraception. To achieve a relative balance of sex in HVTN 100, no more than 60% of trial participants of either sex were enrolled. Baseline Ad26 seropositivity was not exclusionary in HVTN 117/HPX2004.

Both studies were approved by local Institutional Review Boards at the respective participating sites. Both trials were registered at ClinicalTrials.gov (HVTN 100, NCT02404311; HVTN 117/HPX2004, NCT02788045). In addition, HVTN 100 was registered with the South African National Clinical Trials Registry (DOH-27-0215-4796). All participants provided written informed consent in their preferred language.

### 2.3 Study products and vaccination schedule

The ALVAC-HIV (vCP2438) priming vaccine used in HVTN 100 expressed a clade C gp120 Env derived from strain ZM96, linked to the transmembrane anchor (TM) sequence of gp41 (derived from the clade B strain LAI) and Gag and Pro derived from the clade B strain LAI. ALVAC-HIV was administered at a dose of 10^7^ 50% cell culture infectious dose (CCID50) and was donated by Sanofi Pasteur (Swiftwater, PA). The Env protein booster vaccine used in HVTN 100 was comprised of a bivalent clade C gp120 Env derived from strains TV1.C and 1086.C, each at a dose of 100 μg, and was administered with the MF59 oil-in-water emulsion adjuvant provided by GSK (Rixensart, Belgium), formerly Novartis (Cambridge, MA). ALVAC-HIV (vCP2438) was administered at months 0 and 1, and ALVAC-HIV (vCP2438) plus bivalent clade C gp120 with MF59 at months 3, 6 and 12 (week 52; Table A1 in S1 Text).

The HVTN 117/HPX2004 priming vaccine, Ad26.Mos4.HIV, is a replication-deficient adenovirus serotype 26 vector [28] that delivers mosaic Gag, Pol, and Env immunogens [15] and was administered at a dose of 5x10^10^ viral particles (vp) per 0.5 mL injection. The trimeric clade C gp140 vaccination contained 250 μg total protein mixed with 0.425 mg aluminum phosphate (alum) adjuvant per 0.5 mL injection. Vaccinations were given on a schedule of Ad26.Mos4.HIV at month 0 and month 3, and Ad26.Mos4.HIV plus clade C gp140 with alum at months 6 and 12 (week 48; Table SA2 in S1 Text).

### 2.4 Laboratory assays

All immunogenicity assays were performed in a blinded fashion under Good Clinical Laboratory Practice conditions.

#### 2.4.1 Binding antibody responses.

HIV-1 binding antibody multiplex assay (BAMA) was used to measure serum HIV-1 total IgG and specific IgG3 binding antibody responses at the 1:50 and 1:40 dilution, respectively [7,29,30]. Further details about responses against single antigens, including positivity criteria are included in the S1 Text. Additionally, an individual was considered a responder to a given panel if they had a positive response to any of the antigens within that panel, and the magnitude of the responses to a given panel of antigens were summarized with a breadth score. The breadth score is computed as the area under the Magnitude-Breadth (MB) curve for each vaccine recipient and calculated as the average of the log10(net MFI) for each antigen in the panel [31].

#### 2.4.2 T-cell responses.

CD4+ and CD8+ T-cell responses to HIV-1 vaccine insert-matched peptides were measured by intracellular cytokine staining (ICS) [32]. Response calls are only defined for the qualified cellular endpoint: T-cells expressing interleukin-2 (IL-2) and/or interferon-γ (IFN-γ). Additional details, including positivity criteria and a list of antigens, can be found in the S1 Text. To explore polyfunctional cellular responses not captured by the qualified endpoint, Combinatorial Polyfunctionality Analysis of Antigen-Specific T-cell Subsets (COMPASS) was also used to analyze Both Env- and Gag-specific T-cell polyfunctional responses [26]. The COMPASS-derived functionality score (FS) is defined as the estimated proportion of Env-specific (or Gag-specific) subsets detected among all possible subsets. The COMPASS-derived polyfunctionality score (PFS) is similar to the FS, but weights subsets by the degree of functionality, placing larger weights on subsets with higher degrees of function.

### 2.5 Analysis cohort

Analyses were based on the per-protocol (PP) cohorts of both HVTN 100 Part A and HVTN 117/HPX2004, comprised of those study participants who received the first four vaccines as scheduled. All post-acquisition samples from HVTN 100 participants who acquired HIV while on-study were excluded from this analysis; no participants acquired HIV in HVTN 117/HPX2004.

### 2.6 Outcomes

**Fig 1:**
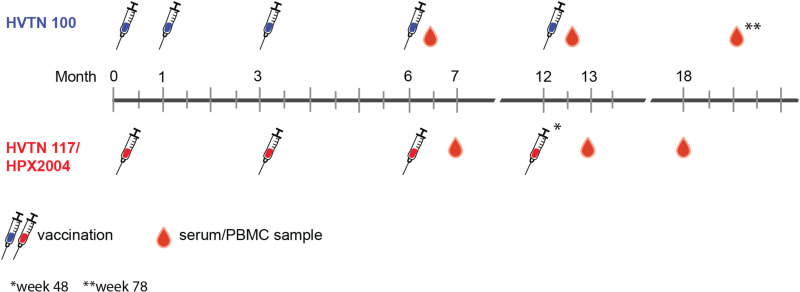
Summary of HVTN 100 and HVTN 117/HPX2004 vaccination and specimen collection schedules. Samples were collected 2 weeks after each vaccination in HVTN 100 and 4 weeks after each vaccination in HVTN 117/HPX2004. The month 12 vaccination was given at week 52 in HVTN 100 and week 48 in HVTN 117/HPX2004. Month 18 sample collections occurred at week 72 in HVTN 100 and week 78 in HVTN 117/HPX2004.

For HVTN 100, where samples were collected two weeks after vaccination, we include results from month 6.5, month 12.5, and month 18; for HVTN 117/HPX2004, where samples were collected 4 weeks post vaccination, the month 7, month 13, and month 18 results are included. Month 18 sample collections occurred at week 72 in HVTN 100 [33] and week 78 in HVTN 117/HPX2004. Cellular and antibody responses were compared from specimens collected at three time points: after the month 6 and month 12 vaccinations (month 6.5/7 and month 12.5/13), as well as six months following the last vaccination (month 18). Fig 1 summarizes the vaccination and sampling schedules used in this analysis. In HVTN 100 Part A, the month 12.5 and 18 binding antibody assays were run on a subset of 65 vaccine recipients stratified by sex assigned at birth to match the ratio in HVTN 100. The HVTN 100 T-cell ICS immune responses were run on a randomly selected subset of 70 vaccine and 5 placebo recipients stratified by sex assigned at birth to match the ratio in HVTN 100.

Primary antibody endpoints were IgG responses to vaccine mismatched gp140, gp120, and V1V2 clade B and clade C antigen panels; IgG responses to the V1V2 RV144 correlate gp70- BCaseA; and IgG3 responses to Con 6 gp120/B, Con S gp140 CFI, and gp70-BCaseA V1V2. The primary cellular endpoints were responses to T-cells expressing interleukin-2 (IL-2) and/or interferon-γ (IFN-γ) and COMPASS-derived polyfunctionality scores to vaccine-matched HIV-1 “Any Env” and “Any Gag” peptide pools. Secondary analyses compared the magnitudes of T-cell responses in the cellular subsets identified by COMPASS at month 12.5/13.

### 2.7 Statistical analysis

The primary analysis used super learning and targeted minimum loss estimation (TMLE) [34] to estimate and compare the mean response rates and mean magnitudes, adjusted for possible confounding by sex assigned at birth, age, and body mass index (BMI) (see S1 Text). Covariate-unadjusted comparisons of immune response rates and magnitudes between the two vaccine groups of interest in HVTN 100 and HVTN 117/HPX2004 were also done using Barnard’s test and Wilcoxon rank-sum tests. These tests were also used to compare covariates between trials. TMLE was not performed on binary endpoints for which the response rates in both trials were over 90% or under 10%, due to lack of variability in the endpoint. TMLE was not performed on continuous endpoints when response rates were less than 10% for only one trial.

All p-values are two-sided and values less than 0.05 were deemed significant. No adjustments were made for multiplicity of hypothesis testing. We report the TMLE estimates, 95% CIs, and p-values throughout the manuscript unless the TMLE results are unavailable. When TMLE was not performed, covariate-unadjusted results are reported and indicated in the text. Both SAS (version 9.4; SAS Institute, Cary, NC, USA) and R statistical software (version 4.0.4; R Foundation for Statistical Computing, Vienna, Austria) were used for statistical analysis.

## 3 Results

### 3.1 Study Populations

Between February 2015 and May 2015, HVTN 100 enrolled 252 participants from South Africa, of whom 210 were allocated to vaccine and 42 to placebo. Between July 2016 and April 2017, HVTN 117/HPX2004 enrolled 181 participants from the United States of America and 17 from Rwanda (total 198), of whom 110 were randomized to the study arm (Group 2A) with the Ad26.Mos4.HIV priming vector. Our analyses compare the per-protocol cohorts from Group 2A of HVTN 117/HPX2004 to the HVTN 100 vaccine arm (Table A in S1 Text) to assess the different immune profiles induced by these distinct viral prime and protein boost vaccine regimens. Among those enrolled, 186 (89%) individuals were in the HVTN 100 vaccine per-protocol cohort; 99 (90%) individuals were in the HVTN 117/HPX2004 tetravalent vaccine per-protocol cohort. While there was no significant difference in sex assigned at birth between the two trials, the trials were conducted in different countries and significant differences were observed in distribution of age and BMI: the median age was 23 years in HVTN 100 versus 28 years in HVTN 117/HPX2004 (Wilcoxon p-value <0.001) and the median BMI was 23 kg/m^2^ in the HVTN 100 vaccine group compared to 25 kg/m^2^ in the HVTN 117/HPX2004 vaccine group (p-value=0.003) (Table 1).

**Table 1:** Baseline characteristics of the per-protocol cohorts of HVTN 100 and HVTN 117/HPX2004 vaccine recipients.

Characteristic		HVTN 100	HVTN 117/HPX2004
		N (%) or	N (%) or
		Median (25%, 75%)	Median (25%, 75%)
**N**		186 (100%)	99 (100%)
**Country**	**South Africa**	186 (100%)	0 (0%)
	**United States**	0 (0%)	89 (90%)
	**Rwanda**	0 (0%)	10 (10%)
**Sex**	**Assigned male at birth**	112 (60%)	52 (53%)
**BMI**	**<18.5**	20 (11%)	4 (4%)
	**18.5-24.99**	104 (56%)	42 (42%)
	**25-29.99**	35 (19%)	28 (28%)
	**>= 30**	26 (14%)	25 (25%)
	**Overall**	23 (20, 27)	25 (22, 30)
**Age (years)**		23 (21, 27)	28 (24, 36)

Vaccine recipients in HVTN 100 are younger and have a lower BMI compared to vaccine recipients of HVTN 117/HPX2004 (Wilcoxon rank sum p-values <0.001 and 0.0003, respectively). The per-protocol cohorts represent 186/210 (89%) vaccine recipients in HVTN 100 and 99/110 (90%) of the vaccine recipients in HVTN 117/HPX2004.

### 3.2 IgG HIV-1 Env binding antibody responses

**Fig 2:**
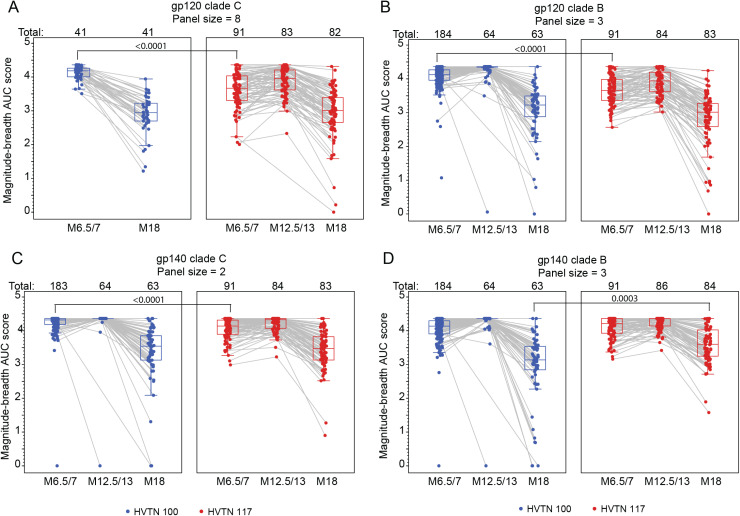
Breadth of IgG binding antibody responses among per-protocol vaccine recipients to the (A) gp120 clade C, (B) gp120 clade B, (C) gp140 clade C, and (D) gp140 clade B antigen panels over time. Each point represents the area under the magnitude-breadth curve for an individual vaccine recipient at each time point. Boxplots summarize the distribution of responses. Gray lines connect observations from the same study participant. Antigen panel details can be found in Supplementary Material; no antigens are vaccine matched. Note: in HVTN 100, participant samples from month 6.5 and month 18 were run for the gp120 clade C panel on a subset of per-protocol participants and no samples from HVTN 100 at month 12.5 were tested against the gp120 clade C panel. Along the top of each panel, Total represents the number of study participants analyzed at each time point. TMLE p-values are presented.

The breadth of IgG responses to HIV-1 Env antigens was summarized by the responses to four antigen panels: gp120 clade B, gp120 clade C, gp140 clade B, and gp140 clade C. Response rates and magnitudes are summarized in Table E1-4 in S1 Text; IgG breadth score trajectories are plotted in Fig 2; and comparisons between the two groups are summarized in Fig A in S1 Text. IgG response rates were high in both HVTN 100 and HVTN 117/HPX2004 vaccine recipients against antigens in the four breadth panels at all three time points, with response rates of at least 88% (Table E1-4 in S1 Text). The only significant difference in response rates was observed in the IgG gp140 clade B breadth panel at month 18, where the 94% (covariate unadjusted 95% CI: 85% - 98%) response rate observed in HVTN 100 was lower compared to the 100% response rate in HVTN 117/HPX2004 (covariate unadjusted 95% CI: 96% -100%; covariate unadjusted p = 0.0204); however, with such high rates of response in both trials, this is not likely clinically meaningful (Fig A in S1 Text).

Month 6.5/7 IgG breadth scores to both gp120 panels and the gp140 clade C panel were significantly higher in HVTN 100 (Table E1-3 in S1 Text; Fig A-C in S1 Text). For example, at month 6.5/7, the estimated IgG gp120 clade C breadth score was 4.10 in HVTN 100 (95% CI: 4.04 to 4.15) and significantly higher than the HVTN 117/HPX2004 breadth score of 3.61 (95% CI: 3.51 to 3.72; TMLE p=<0.0001; Table E1 in S1 Text; Fig C in S1 Text). This early difference is perhaps attributable to the additional vaccination administered at month 1 in HVTN 100. However, at Month 12.5/13, the distribution of IgG breadth scores to gp120/gp140 was very high in both studies, with breadth scores clustered near the upper limit of the assay; no significant differences were observed between the two trials (Table E2-4 in S1 Text). Six months following the last vaccination, at month 18, the breadth of IgG responses was similarly durable for both gp120 panels and the gp140 clade C panel, with higher durability observed in HVTN 117/HPX2004 for the gp140 clade B panel (2.89 (2.53, 3.24) in HVTN 100 vs 3.58 (3.47, 3.69) in HVTN 117/HPX2004; TMLE p-value=0.0003; Table E4 in S1 Text; Fig B in S1 Text).

Only 1 positive response to gp41 IgG was observed in HVTN 100 at each analysis time point (0.5% and 1.6% at month 6.5/7 and month 12.5/13, respectively); in contrast, 100% of HVTN 117/HPX2004 vaccine recipients had positive responses at both time points (Fig E in S1 Text; Table E8 in S1 Text).

### 3.3 IgG V1V2 binding antibody responses

**Fig 3:**
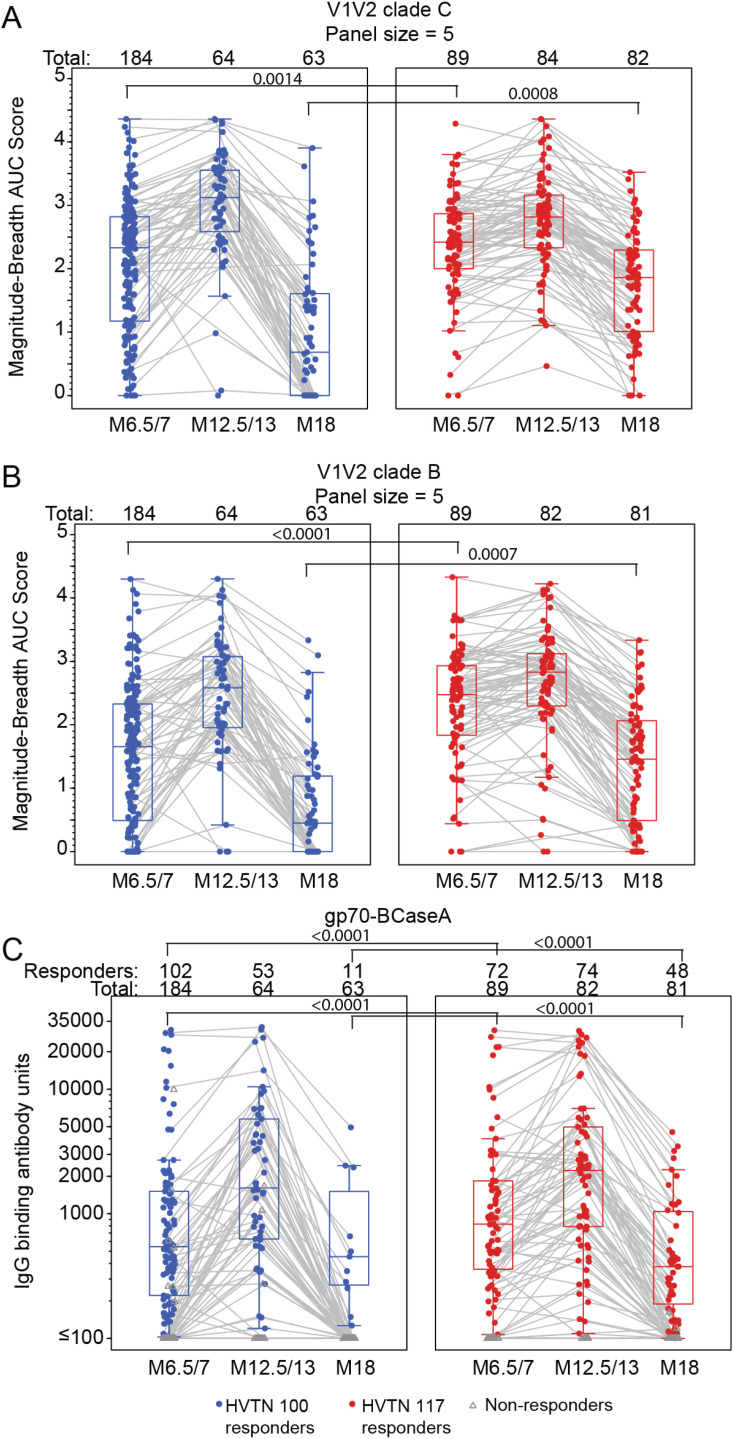
Breadth of IgG binding antibody responses to the V1V2 clade C (A) and clade B (B) antigen panels and summary of IgG responses to V1V2 RV144 correlate gp70-BCaseA (C) Each point represents the area under the magnitude-breadth curve for an individual vaccine recipient at each time point. Boxplots summarize distribution of responses and gray lines connect observations from the same study participant. Note: HVTN 100 samples were only run on a subset of 65 per-protocol participants at months 12.5 and 18. Antigen panel details can be found in Supplementary Material, and no antigens are vaccine matched. For the single antigen in (C), boxplots summarize the distribution of positive responses (shown as colored circles), negative responders are shown as gray triangles. Gray lines connect observations from the same study participant. Binding antibody units are net MFI. Along the top of each panel, Total represents the number of study participants analyzed at each time point, and Responders the number of those who had a positive response. TMLE p-values are presented.

Although IgG response rates and magnitude against both the V1V2 clade B and clade C breadth panels were similar at month 12.5/13, responses to both panels were significantly lower in HVTN 100 compared to HVTN 117/HPX2004 at months 6.5/7 and 18 (Fig 3A-B, Table E5,6 in S1 Text, Fig A in S1 Text, Fig D in S1 Text). At month 6.5/7 in HVTN 100, 76% responded to at least one antigen in the V1V2 clade C panel, compared to 90% in HVTN 117/HPX2004 (TMLE p = 0.0026; Table E5 in S1 Text); the estimated mean V1V2 clade C breadth score at this timepoint was 2.01 in HVTN 100 (TMLE 95% CI: 1.84 to 2.17), significantly lower than the HVTN 117 breadth score of 2.38 (TMLE 95% CI: 2.22 to 2.53; TMLE p=0.0014; Table E5 in S1 Text). Similarly, for the V1V2 clade B panel at month 6.5/7, 60.0% (TMLE 95% CI: 53% - 67%) of HVTN 100 participants responded compared to the 87.0% in HVTN 117/HPX2004 (TMLE 95% CI: 80% - 94%; TMLE p < 0.0001; Table E6 in S1 Text), and the estimated mean V1V2 clade B breadth score was 1.54 in HVTN 100 (TMLE 95% CI: 1.38 to 1.7) and 2.33 in HVTN 117 (TMLE 95% CI: 2.15 to 2.51; TMLE p < 0.0001) (Fig 3B).

Six months following the last vaccination, the IgG response rate to the V1V2 clade C panel remained significantly lower in HVTN 100 (40%; TMLE 95% CI: 26% - 55%) compared to HVTN 117/HPX2004 (68%; TMLE 95% CI: 58% - 78%; TMLE p=0.0025; Table E5 in S1 Text). V1V2 clade B IgG response rates were also significantly lower in HVTN 100 compared to HVTN 117/HPX2004 (24.2% vs 66.7%, TMLE p < 0.0001; Table E6 in S1 Text) at month 18.

IgG response rates to the gp70-BCaseA V1V2 antigen (a primary correlate of risk in RV144) were consistently lower in HVTN 100 compared to HVTN 117/HPX2004 across all three time points, with significant differences in response rates at months 6.5/7 and 18 (Fig 3C; Table E7 in S1 Text). At month 6.5/7, 54% (TMLE 95% CI: 46% - 61%) of HVTN 100 participants had a positive IgG response compared to 82% of HVTN 117/HPX2004 participants (TMLE 95% CI: 74% - 90%; TMLE p<0.0001; Table E7 in S1 Text). Response rates rose in both trials after the last vaccination, with 77% (TMLE 95% CI: 65%-89%) of HVTN 100 and 90% of HVTN 117/HPX2004 participants responding at month 12.5/13 (TMLE 95% CI: 83%-97%; TMLE p=0.0573; Table E7 in S1 Text). By month 18, the response rate was 18% (TMLE 95% CI: 7%-29%) for HVTN 100 versus 62% in HVTN 117/HPX2004 (TMLE 95% CI: 50% - 73%; TMLE p < 0.0001; Table E7 in S1 Text). As a result of the differences in response rates to the gp70-BCaseA V1V2 antigen, IgG magnitudes overall were found to be significantly lower in HVTN 100 at month 6.5/7 and month 18. However, among the subset of positive responders no significant differences in the magnitude of IgG responses were observed at any timepoint (Fig 3C, Table E7 in S1 Text).

### 3.4 IgG3 HIV-1 Env binding antibody responses

**Fig 4:**
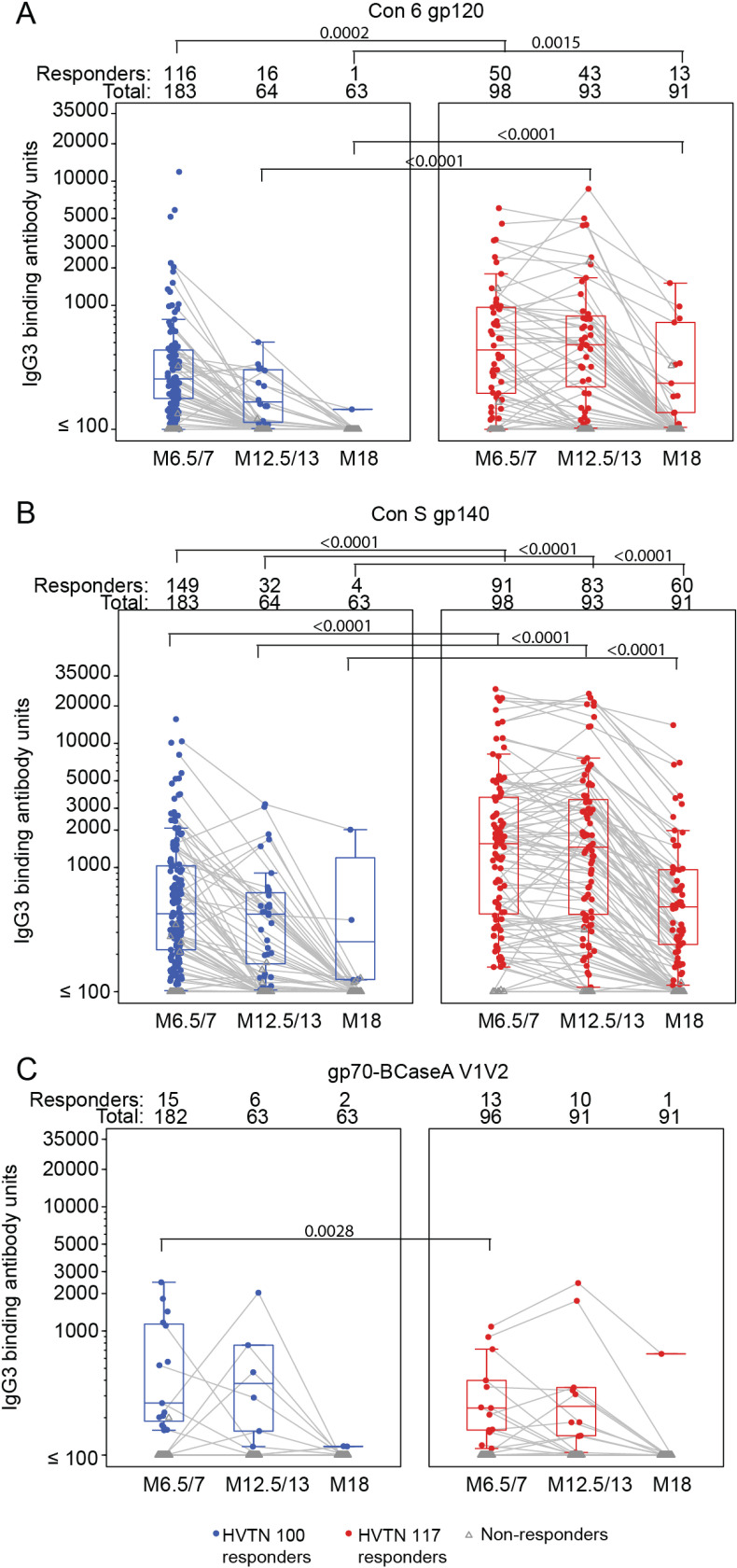
Summary of IgG3 binding antibody responses to Con 6 gp120/B (A), Con S gp140 CFI (B), and gp70-BCaseA V1V2 (C) antigens. Boxplots summarize the magnitude of IgG3 responses among positive responders (shown as colored circles), negative responders are shown as gray triangles. Gray lines connect observations from the same study participant. Binding antibody units are net MFI. Note: HVTN 100 samples were only run on a subset of 65 per-protocol participants at months 12.5 and 18. Along the top of each panel, Total represents the number of study participants analyzed at each time point, and Responders the number of those who had a positive response. TMLE p-values are presented.

IgG3 response rates to Con 6 gp120B were highest at month 6.5/7, where 62% of the 183 HVTN 100 participants had a positive IgG3 response to the consensus gp120 (TMLE 95% CI: 55% - 69%) compared to 54% of HVTN 117/HPX2004 participants (TMLE 95% CI: 43% - 65%; TMLE p-value=0.21; Fig 4A; Table E9 in S1 Text). IgG3 response rates dropped after subsequent vaccination in both trials. In HVTN 100, only 21% of the 64 participants had an IgG3 response at month 12.5/13 (TMLE 95% CI: 11% - 30%), significantly lower than the 47% response rate seen in HVTN 117/HPX2004 (TMLE 95% CI: 36% - 58%, TMLE p = 0.0002; Table E9 in S1 Text).While the geometric mean responses were similar in the two regimens at month 6.5/7, the geometric mean response in HVTN 117/HPX2004 was 4.4-fold higher than that observed in HVTN 100 at month 12.5/13 (TMLE p<0.0001; Table E9 in S1 Text).

While IgG3 responses to Con S gp140 decreased over subsequent vaccinations, response rates and magnitudes were significantly higher in HVTN 117/HPX2004 compared to HVTN 100 at all three times (Table E10 in S1 Text). Among HVTN 100 participants, IgG3 response rates to Con S gp140 declined from 81% at month 6.5/7 (TMLE 95% CI: 75% - 87%) to 42% (TMLE 95% CI: 29%-55%) at month 12.5/13, despite the subsequent vaccination (Fig 4B; Table E10 in S1 Text). In contrast, the IgG3 response rate to Con S gp140 among HVTN 117/HPX2004 Mos4 recipients was 95% (TMLE 95% CI: 91%-99%) at month 6.5/7, and after the last vaccination was 89% (TMLE 95% CI: 82%-96%). The IgG3 response rate to Con S gp140 was significantly higher in HVTN 117/HPX2004 than in HVTN 100 (both month 6.5/7 and 12.5/13 TMLE p<0.0001; Table E10 in S1 Text). Moreover, the geometric mean IgG3 response in HVTN 117/HPX2004 was 3.8 times higher than that observed in HVTN 100 at month 6.5/7, and 9.4 times higher at month 12.5/13 (each TMLE p<0.0001; Fig A in S1 Text, Table E10 in S1 Text).

IgG3 response rates to gp70-BCaseA V1V2 were low in both trials and not significantly different (Fig 4C; Table E11 in S1 Text). At month 6.5/7, only 7% (TMLE 95% CI: 3%-10%) of HVTN 100 participants had a positive response, compared to 15% (TMLE 95% CI: 6%-23%) response rate observed in the HVTN 117/HPX2004 vaccine group (TMLE p-value=0.091; Table E11 in S1 Text). After the last vaccination, 9.6% (TMLE 95% CI: 1%-18%) of HVTN 100 participants were observed to have a positive IgG3 response to gp70-BCaseA V1V2, compared to 10% (TMLE 95% CI: 4%-17%) of HVTN 117/HPX2004 participants (TMLE p-value=0.89; Table E11 in S1 Text).

Finally, significantly higher IgG3 responses against gp41 were observed in HVTN 117/HPX2004, with response rates of at least 85% in HVTN 117/HPX2004, while no responses were observed in HVTN 100 (Fig E in S1 Text; Table E12 in S1 Text).

### 3.5 CD4+ T-cell responses

**Fig 5:**
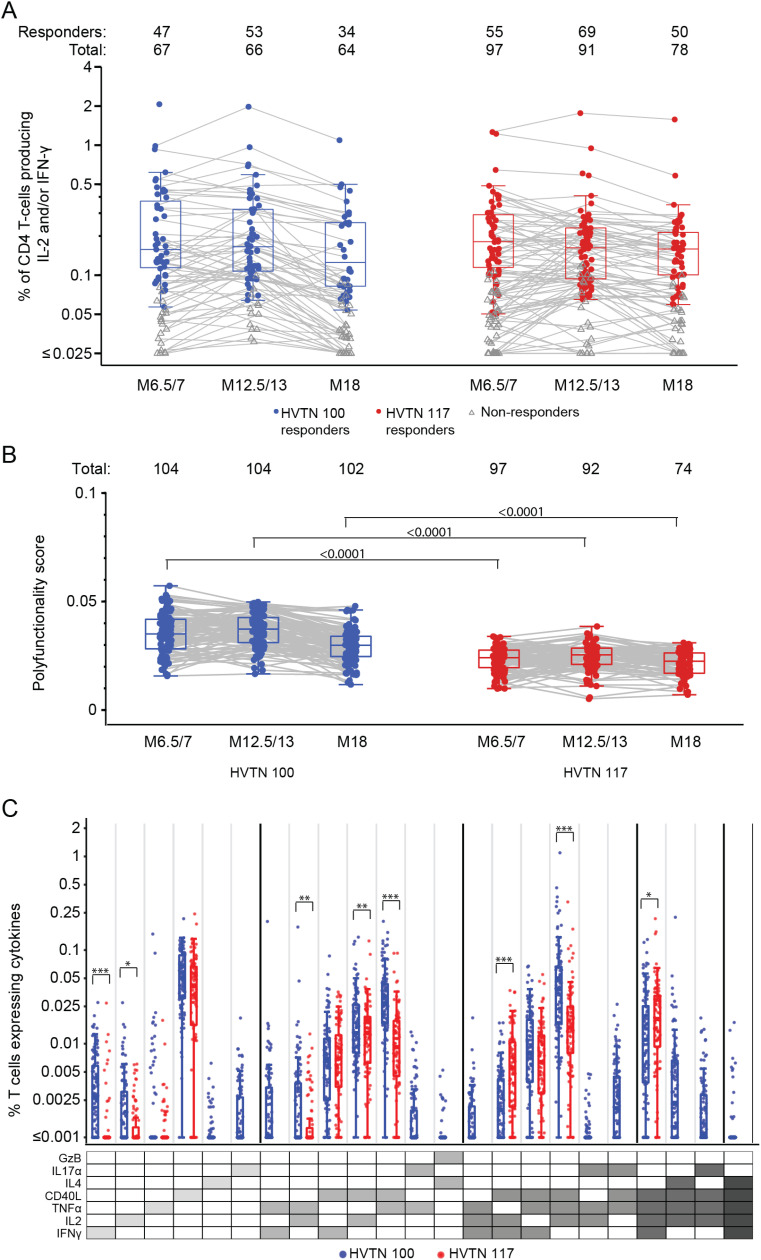
Summary of CD4+ T-cell vaccine-matched Env peptides to (A) IL-2 and/or IFN-γ and (B) polyfunctionality scores over time, and (C) magnitudes of marker-specific CD4 + T-cell responses at month 12.5/13. Boxplots showing IL-2 and/or IFN-γ responses and are based on positive responders only (shown as colored circles), negative responders are shown as gray triangles. Gray lines connect observations from the same study participant. Boxplots showing the CD4+ T-cell polyfunctionality scores for all participants, stratified by protocol. Lines connect scores for the same participant. Along the top of each panel, Total represents the number of study participants analyzed at each time point, and Responders the number of those who had a positive response. Boxplots summarize subset specific CD4+ T-cell responses at Month 12.5/13 for all participants, ordered according to the cytokine legend in gray where cellular subsets are denoted by the cytokines they express (white, “off”; shaded “on”) and ordered from single function to higher-degree functionality. Cytokine combinations with COMPASS-estimated average posterior probabilities of at least 0.005 in either protocol are shown; if a subset has only one protocol plotted, then the subset was only identified in that protocol. TMLE p values are shown. In part C, * is p<0.05; ** is p<0.01; and *** is p<0.001.

Similar response rates and magnitudes for CD4+ T cells expressing interleukin-2 (IL-2) and/or interferon-γ (IFN-γ) to any vaccine-matched Env peptide were observed in both HVTN 100 and HVTN 117/HPX2004 vaccine recipients at all time points (Fig 5A; Table E13 in S1 Text). However, when considering the broader polyfunctional response, significant differences were seen in Env-specific CD4+ polyfunctionality scores (PFSs) (Fig 5B; Table E17 in S1 Text). At each timepoint, PFSs were significantly higher in HVTN 100 vaccine recipients compared to those enrolled in HVTN 117/HPX2004 (TMLE p<0.0001 at each analysis time; Table E17 in S1 Text). For example, after the last vaccination the covariate adjusted mean PFS in HVTN 100 was 0.033 (TMLE 95% CI: 0.031 to 0.035), significantly higher than the estimated mean PFS of 0.023 seen in HVTN 117/HPX2004 (TMLE 95% CI: 0.022 to 0.024; TMLE p < 0.0001; Table E17 in S1 Text). As higher PFS could be the result of inducing more functional subsets or subsets of a higher degree of functionality, the observed differences in CD4+ T-cell polyfunctionality motivated a post-hoc exploratory analysis of the responses in the functional subsets.

To further to understand the differential expression of specific CD4+ T cell subsets generated by these two vaccine regimens, we compared the magnitude of responses across the cytokine subsets identified by the COMPASS analyses at month 12.5/13 (Fig 5C, Table F in S1 Text). HVTN 100 generated higher magnitudes of CD4+ T cell to Env responses for several marker subsets. At month 12.5/13, the average covariate-adjusted magnitude of Env-specific CD4+ T cells expressing TNF-α and CD40L was estimated to be 0.028% (TMLE 95% CI: 0.024% – 0.033%) in HVTN 100 significantly higher than that of HVTN 117/HPX2004, which was 0.008% (TMLE 95% CI: 0.006% – 0.01%, TMLE p-value <0.001; Table F in S1 Text). The average magnitude of Env-specific CD4+ T cells expressing IL-2, TNF-α, and CD40L at month 12.5/13, was 0.04% (TMLE 95% CI: 0.031% – 0.051%) in HVTN 100 and significantly higher than that of HVTN 117/HPX2004 which was 0.013% (TMLE 95% CI: 0.01% – 0.017%, TMLE p-value <0.001). Together, these results suggest HVTN 100 not only generated CD4+ Env responses to a broader range of cytokine subsets, but also responses were seen in subsets with higher degrees of functionality compared to HVTN 117/HPX2004.

While significantly higher polyfunctionality CD4+ Gag responses were observed in HVTN 117/HPX2004, the magnitudes of responses to IL-2 and/or IFN-γ and additional cytokine subsets were similar between the two protocols (Fig A,F in S1 Text; Table E14,17 in S1 Text).

### 3.6 CD8+ T-cell responses

**Fig 6:**
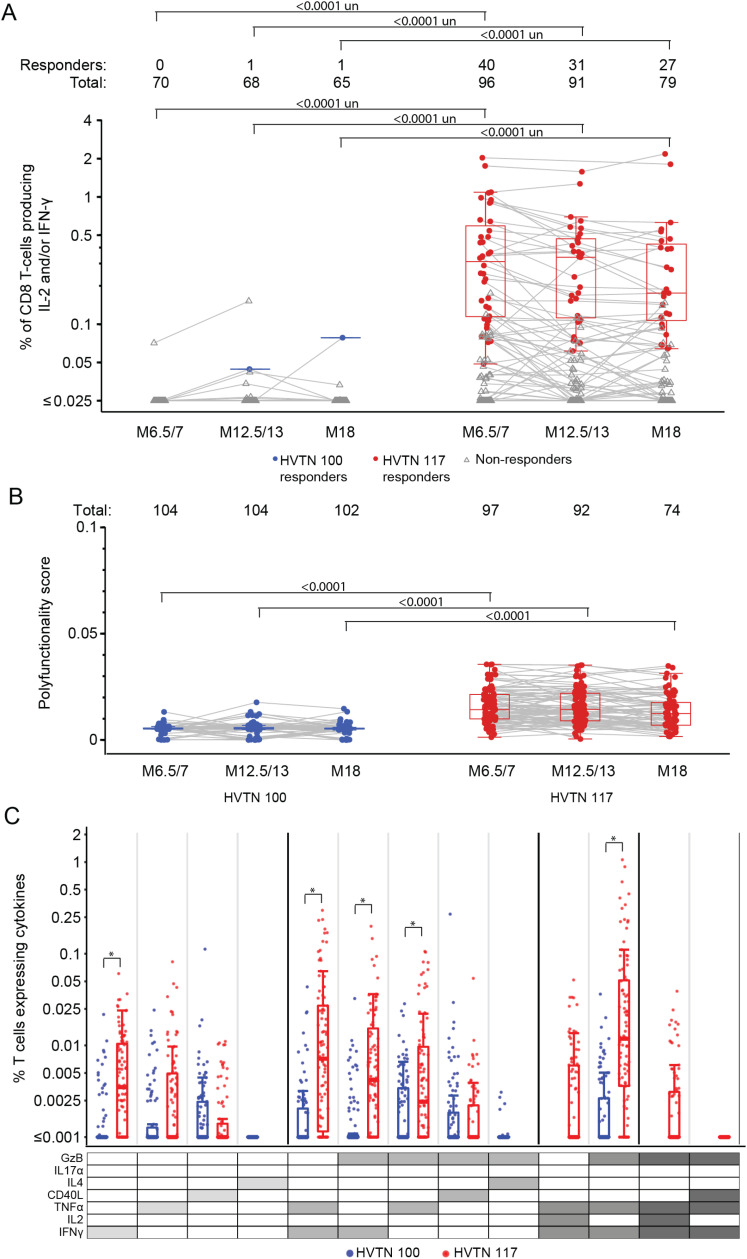
Summary of CD8+ T-cell vaccine-matched Env peptides to (A) IL-2 and/or IFN-γ and (B) polyfunctionality scores over time, and (C) magnitudes of marker-specific CD8 + T-cell responses at month 12.5/13. Boxplots showing IL-2 and/or IFN-γ responses and are based on positive responders only (shown as colored circles), negative responders are shown as gray triangles. Gray lines connect observations from the same study participant. Boxplots showing the CD8+ T-cell polyfunctionality scores for all participants, stratified by protocol. Lines connect scores for the same participant. Along the top panels A and B, Total represents the number of study participants analyzed at each time point, and Responders the number of those who had a positive response. Boxplots summarize subset specific CD8+ T-cell responses at Month 12.5/13 for all participants, ordered according to the cytokine legend in gray where cellular subsets are denoted by the cytokines they express (white, “off”; shaded “on”) and ordered from single function to higher-degree functionality. Cytokine combinations with COMPASS-estimated average posterior probabilities of at least 0.005 for either protocol are shown. TMLE p values are shown. In part A, un=unadjusted p values. In part C, * is p<0.001.

HIV-specific CD8+ T cell responses were almost exclusively observed in HVTN 117/HPX2004. None of the 70 HVTN 100 participants had a positive CD8+ response to any vaccine-matched Env peptide at month 6.5/7 (covariate unadjusted 95% CI: 0% - 5.2%), and only 1 participant was observed to have a positive response at either of the subsequent time points (Fig 6A; Table E15 in S1 Text). In contrast, after the month 6 vaccination, 42% (covariate unadjusted 95% CI: 32%-52%) of HVTN 117/HPX2004 participants had CD8+ T cells expressing IL-2 and/or IFN-γ to at least one of the vaccine-matched Env peptide pools; at month 12.5/13, the response rate was 34% (covariate unadjusted 95% CI: 25% - 44%); and 34% (covariate unadjusted 95% CI: 25% -45%) at month 18 (Table E15 in S1 Text). The difference in response rate was significant for each timepoint (covariate unadjusted p < 0.0001; Table E15 in S1 Text).

A similar pattern was seen for CD8+ T cell responses to any Gag peptide pool (Fig A,G in S1 Text; Table E16 in S1 Text). Responses for CD8+ T cells expressing IL-2 and/or IFN-γ to any vaccine-matched Gag peptide pool were only observed in HVTN 117/HPX2004, where 32% (covariate unadjusted 95% CI: 23% - 42%; Table E16 in S1 Text) were observed to have a positive response at month 12.5/13 (Fig G in S1 Text). Moreover, the CD8+ T cell responses to Any Gag was durable, with 33% (covariate unadjusted 95% CI: 24% - 44%; Table E16 in S1 Text) of HVTN 117/HPX2004 vaccine recipients positive at month 18.

Additionally, CD8+ T-cell polyfunctionality to both Env and Gag peptides were significantly higher among HVTN 117/HPX2004 vaccine recipients compared to those in HVTN 100 after each vaccination (TMLE p<0.0001 at each time point; Fig 6B, Table E17 in S1 Text, Fig G in S1 Text). Post-hoc analysis of the magnitudes of functional marker subset responses (Fig 6C) found higher median magnitudes in HVTN 117/HPX2004 compared to those in HVTN 100 for most functional subsets identified by the polyfunctionality analysis. While the HVTN 100 responses tended to be significantly lower than those in HVTN 117/HPX2004, similar magnitudes between the two trials were seen in specific rare subsets. For example, the mean response to CD8+ T-cells expressing only CD40L was 0.002% in HVTN 100 (covariate unadjusted 95% CI: 0.000% - 0.007%) and higher than the mean magnitude of 0.001% seen to HVTN 117/HPX2004, although not statistically significant (95% CI: 0.000% - 0.006%; covariate unadjusted p=0.167; Table G in S1 Text). Similar magnitudes of responses were seen in marker subsets expressing TNF-α and GzB together, and CD40L and GzB together (Table G in S1 Text). These specific subsets are not captured in the IL-2 and/or IFN-γ endpoint and were of low magnitude. Interestingly, HVTN 117/HPX2004 was seen to induce high magnitudes of the highly polyfunctional Env-specific CD8+ T cells expressing GzB, TNF-α, and IFN-γ together with a mean magnitude of 0.014% (95% CI: 0.0% to 0.553%; Table G in S1 Text).

## 4 Discussion

Two recent vaccine efficacy trials found no statistically significant evidence of vaccine efficacy against HIV-1 acquisition (point estimates of approximately 0% in HVTN 702 (AFAB and AMAB) and 14.1% in HVTN 705/HPX2008 (AFAB only)). Using the early phase trial data, we wanted to understand whether both inefficacious vaccines are characterized by a similar versus different pattern of immune responses, to guide the field to better understand unfavorable vaccine immunogenicity patterns that should not be targeted with future vaccine regimens. We compared vaccine-induced immune responses at three timepoints from two vaccine regimens from different platforms: clade B/C canarypox vector Gag/Env insert prime (ALVAC-HIV (vCP2438)) with an ALVAC-HIV + clade C Env gp120/MF59 boost (HVTN 100) and tetravalent adenovirus serotype 26 vector mosaic Gag/Pol/Env inserts prime (Ad26.Mos4.HIV) with Ad26.Mos4.HIV + trimeric subtype C gp140/alum boost (HVTN 117/HPX2004). Our analysis focused on previously identified antibody and T-cell correlates of protection [7,9,24,35].

V1V2 Env binding antibody responses were identified as correlates of risk in both RV144 and HVTN 702 [24,27] and thus were of primary interest in our comparative analysis. We saw that despite the two regimens achieving similar levels at month 12.5/13, HVTN 117/HPX2004 elicited higher IgG V1V2 responses at month 6.5/7 and more durable responses by month 18. Taken together, this analysis suggests that the earlier and more sustained IgG V1V2 responses seen in HVTN 117/HPX2004 may explain a part of the modest potential protection seen in HVTN 705/HPX2008 [35]. Additionally, both trials elicited low levels of IgG3 V1V2 responses, consistent with the HVTN 705/HPX2008 finding that that the rare subgroup of vaccine recipients with outlying high IgG3 V1V2 responses received partial protection from the vaccine [35].

We also compared the T-cell responses induced by the two regimens, as previous analyses have suggested that T-cell responses influence HIV-1 acquisition risk [7,24,36]. Consistently higher polyfunctional CD4+ Env T cells were seen in HVTN 100 compared to HVTN 117, although the response rates and magnitudes of CD4+ T cells expressing IFN-γ and/or IL-2 to vaccine-matched HIV-1 Env were similar. While CD4+ polyfunctionality scores to envelope peptides were found to be an inverse correlate of HIV-1 risk in RV144, the HVTN 702 correlates analysis found that the risk of HIV-1 acquisition depended on CD4+ polyfunctionality scores combined with IgG A244 V1V2 levels [24]. This relationship could not be evaluated in the current analysis and was not evaluated in the HVTN 705/HPX2008 correlates analysis.

Finally, several immune responses were seen only among HVTN 117/HPX2004 participants: IgG gp41; IgG3 gp41; CD8+ T cells expressing IFN-γ and/or IL-2 to vaccine-matched HIV-1 Env and Gag peptide pools; and polyfunctional Env and Gag CD8+ T-cell responses. These differences are unsurprising, as gp41 was included in the Ad26.Mos4.HIV vaccine in HVTN 117/HPX2004 and the HVTN 117/HPX2004 regimen was designed to induce CD8+ T-cell responses. While a strong inverse correlation between Env-specific CD8+ T-cell responses and HIV-1 acquisition was seen in HVTN 505 [37], the modest lack of efficacy of HVTN 705/HPX2008 suggests that the CD8+ responses induced by Ad26.Mos4.HIV in HVTN 117/HPX2004 were not sufficient for protection.

We conjecture that the inability of the vaccine regimens to induce higher and broader IgG and IgG3 V1V2 antibody levels was a significant factor in the failure of these regimens to prevent HIV-1. The TV1.C and 1086.C gp120 vaccine strains of the HVTN 100 regimen poorly presented V1V2 epitopes accessible to antibodies, especially when delivered with MF59, whereas in contrast the A244 gp120 vaccine strain of the RV144 vaccine regimen delivered in alum was more accessible to V1V2 antibodies [38]. The HVTN 117/HPX2004 gp140 booster strain C97ZA was more accessible to V1V2 antibodies, but for less than 20% of vaccine recipients, with explanation unclear. One clue comes from HVTN 124 of homologous prime-boost with whole Env proteins that elicited high V1V2 antibodies in many more vaccine recipients [39]. Future research could pursue greater explanation of the lack of frequent high IgG and IgG3 V1V2 response phenotype of HVTN 100 and HVTN 117/HPX2004 vaccine recipients, such as through structural biology including sequence-prediction machine learning algorithms [40]. In addition, it is well known no HIV-1 vaccine platform has been able to elicit neutralizing antibodies recognizing exposing viral strains, where neutralization has been established as a correlate of protection for more than a dozen approved vaccines [41].

This study had several limitations. Specimens from HVTN 100 and HVTN 117/HPX2004 were not run on the assays concurrently. While most of the endpoints were measured with qualified assays, these results could still be impacted by variations in the assay over time. Some important endpoints could not be explored in this analysis. ADCC responses were not compared between trials due to changes in the assay between the two trials (laboratory work approximately 3 years apart) that precluded cross-protocol analysis [42]. IgA was not assessed at the same dilution in each trial thus not compared. As no further assays were run, other potentially interesting immunogenicity endpoints were excluded from this analysis. For example, avidity was not assessed in either trial and therefore unavailable for this analysis.

Another limitation is the difference in vaccination schedules: HVTN 100 administered an additional priming vaccination at month 1; thus, differences in immune responses seen at month 6.5 could be partially attributable to the additional priming dose by month 6. Differences in the protocol-specified timing of specimen collection after vaccination (2 weeks in HVTN 100 and 4 weeks in HVTN 117/HPX2004) could also affect our conclusions. Importantly, HVTN 100 and HVTN 117/HPX2004 were conducted in different populations. Although we adjusted for basic demographic differences in the participant populations, we cannot fully disentangle the differences in vaccine-induced immune responses and unmeasured country-specific effects. The studies were conducted in distinct geographic regions where complex sociodemographic and genetic variability may have played a role in the differences seen in immune responses [43].

Despite these limitations, the current analysis provides a direct comparison of humoral and T-cell responses, including previously identified correlates, in the two phase 1/2 trials at multiple time points. Both vaccine regimens induced robust IgG gp120 and gp140 breadth with weaker responses to V1V2, suggesting that strong IgG responses to gp120 and gp140 are not sufficient for protection. Our results are consistent with the hypothesized role of V1V2 antibodies contributing to vaccine protection, provided additional context to the relative nature of these results, and our study also highlighted specific T-cell subsets that drive the differences in polyfunctionality scores. These factors may be critical to understanding how these two vaccine regimens failed to provide protection for the development and evaluation of future HIV-1 vaccine candidates.

## Supporting information

S1 Text Supplementary Material for ALVAC-Prime and Monomeric gp120 Protein Boost Induces Distinct HIV-1 Specific Humoral and Cellular Responses Compared with Adenovirus-Prime and Trimeric gp140 Protein Boost. (DOCX)
