# Supplementary material for: ALVAC-prime and monomeric gp120 protein boost induces distinct HIV-1 specific humoral and cellular responses compared with adenovirus-prime and trimeric gp140 protein boost
Source: PLOS Glob Public Health. 2025 Apr 11;5(4):e0004250. doi: 10.1371/journal.pgph.0004250 (PMC11990749; doi:10.1371/journal.pgph.0004250)
Supplement: S1 Text — (DOCX) [file pgph.0004250.s001.docx]

S1 Text:

Supplemental Materials for “ALVAC-Prime and Monomeric gp120 Protein Boost Induces Distinct HIV-1 Specific Humoral and Cellular Responses Compared with Adenovirus-Prime and Trimeric gp140 Protein Boost”

Table of Contents

[Quick Links to Supplemental Tables and Figures 2](#_Toc191458834)

[Supplemental materials and methods 3](#_Toc191458835)

[SA Table. Summary of Study Schemas 3](#_Toc191458836)

[Additional protocol details 4](#_Toc191458837)

[Randomization and masking 4](#_Toc191458838)

[Binding antibody multiplex assay (BAMA) to measure binding antibody (bAb) response 5](#_Toc191458839)

[Intracellular cytokine staining (ICS) to measure T-cell response 6](#_Toc191458840)

[Statistical Methods 9](#_Toc191458841)

[Supplemental Tables 10](#_Toc191458842)

[Supplemental Figures 31](#_Toc191458843)

# Quick Links to Supplemental Tables and Figures

[**Table E.** **Response rates (95% CIs), mean magnitudes (95% CIs), and mean magnitudes among positive responders (95% Cis) of humoral and cellular responses by unadjusted and adjusted statistical methods.** 10](#_Toc191555511)

[**Table E1. IgG gp120 clade C breadth** 10](#_Toc191555512)

[**Table E2. IgG gp120 clade B breadth** 11](#_Toc191555513)

[**Table E3. IgG gp140 clade C breadth** 12](#_Toc191555514)

[**Table E4. IgG gp140 clade B breadth** 13](#_Toc191555515)

[**Table E5. IgG V1V2 clade C breadth** 14](#_Toc191555516)

[**Table E6. IgG V1V2 clade B breadth** 15](#_Toc191555517)

[**Table E7. IgG gp70-BCaseA V1V2** 16](#_Toc191555518)

[**Table E8. IgG gp41** 17](#_Toc191555519)

[**Table E9. IgG3 Con 6 gp120/B** 18](#_Toc191555520)

[**Table E10. IgG3 Con S gp140 CFI** 19](#_Toc191555521)

[**Table E11. IgG3 gp70 BCaseA V1V2** 20](#_Toc191555522)

[**Table E12. IgG3 gp41** 21](#_Toc191555523)

[**Table E13. CD4+ Env** 22](#_Toc191555524)

[**Table E14. CD4+ Gag** 23](#_Toc191555525)

[**Table E15. CD8+ Env** 24](#_Toc191555526)

[**Table E16. CD8+ Gag** 25](#_Toc191555527)

[**Table E17. CD4+ and CD8+ polyfunctionality scores (PFS) to Env and Gag** 26](#_Toc191555528)

[**Table E18. CD4+ and CD8+ functionality scores (FS) to Env and Gag** 27](#_Toc191555529)

[**Table F. CD4+ responses to COMPASS identified, Env-specific subsets at Month 12.5/13** 28](#_Toc191555530)

[**Table G. CD8+ responses to COMPASS identified, Env-specific subsets at Month 12.5/13** 30](#_Toc191555531)

[**Fig A. Forest plot summarizing the differences in immune responses** 31](#_Toc191555532)

[**Fig B.** **Magnitude-breadth of IgG gp140 Responses.** 32](#_Toc191555533)

[**Fig C.** **Magnitude-breadth of IgG gp120 Responses.** 33](#_Toc191555534)

[**Fig D. Magnitude-breadth of V1V2 IgG Responses.** 34](#_Toc191555535)

[**Fig E. Summary of IgG (A) and IgG3 (B) responses to gp41.** 35](#_Toc191555536)

[**Fig F. Summary of CD4+ T-cell vaccine-matched Gag peptides to (A) IL-2 and/or IFN-γ and (B) polyfunctionality scores over time, and (C) magnitudes of marker-specific CD4+ T-cell responses at Month 12.5/13.** 36](#_Toc191555537)

[**Fig G. Summary of CD8+ T-cell vaccine-matched Gag peptides to (A) IL-2 and/or IFN-γ and (B) polyfunctionality scores over time, and (C) magnitudes of marker-specific CD8+ T-cell responses at Month 12.5/13.** 38](#_Toc191555538)

# Supplemental materials and methods

## Table A. Summary of Study Schemas

**Table A1.** HVTN 100 Study Schema

| Group | N | Primary vaccine regimen | | | | Booster |
| --- | --- | --- | --- | --- | --- | --- |
|  |  | Month 0 | Month 1 | Month 3 | Month 6 | Month 12 (Week 52) |
| 1 | 210 | ALVAC-HIV (vCP2438) | ALVAC-HIV (vCP2438) | ALVAC-HIV (vCP2438) + Bivalent Subtype C gp120/MF59 | ALVAC-HIV (vCP2438)+ Bivalent Subtype C gp120/MF59 | ALVAC-HIV (vCP2438) + Bivalent Subtype C gp120/MF59 |
| 2 | 42 | Placebo | Placebo | Placebo + Placebo | Placebo + Placebo | Placebo + Placebo |
| Total | 252 |  |  |  |  |  |

**Table A2.** TRAVERSE (HVTN 117/HPX2004) Study Schema

| Group | Subgroup | N | Month 0 | Month 3 | Month 6 | Month 12 (Week 48) |
| --- | --- | --- | --- | --- | --- | --- |
| 1 | A | 55 | Ad26.Mos.HIV | Ad26.Mos.HIV | Ad26.Mos.HIV + clade C gp140/alum | Ad26.Mos.HIV + clade C gp140/alum |
|  | B | 11 | Placebo | Placebo | Placebo + Placebo | Placebo + Placebo |
| 2 | A | 110 | Ad26.Mos4.HIV | Ad26.Mos4.HIV | Ad26.Mos4.HIV + clade C gp140/alum | Ad26.Mos4.HIV + clade C gp140/alum |
|  | B | 22 | Placebo | Placebo | Placebo + Placebo | Placebo + Placebo |

## Additional protocol details

Both studies were approved by local Institutional Review Boards at the respective participating clinical research sites. Both trials were registered with the United States National Institutes of Health Clinical Trials Registry (ClinicalTrials.gov: HVTN 100, NCT02404311; and HVTN 117, NCT02788045). In addition, HVTN 100 was registered with the South African National Clinical Trials Registry (DOH-27-0215-4796). All participants provided written informed consent in their preferred language.

## Randomization and masking

Participants were randomly assigned to receive vaccine or placebo in a 5:1 ratio in HVTN 100 Part A (**Table A1**). The statistical center (SCHARP, Seattle, WA, USA) produced the block-randomized sequences for HVTN 100 by computer-generated random numbers, provided to each site through a web-based randomization system.

In HVTN 117/HPX2004, participants were randomized into one of four subgroups: 55 participants to receive vaccination with Ad26.Mos.HIV and clade C gp140 in Subgroup 1A, 11 participants to receive placebo in Subgroup 1B, 110 participants to receive vaccination with Ad26.Mos4.HIV and clade C gp140 in Subgroup 2A (the subgroup included in this analysis) and 22 participants to receive placebo in Subgroup 2B (**Table A2**). Randomization was performed using a computer-generated randomization schedule that was evaluated by using randomly permuted blocks and stratified by region. A centralized interactive web response system (IWRS) assigned a unique treatment code dictating the treatment assignment and matching study vaccine for each participant.

Participants, clinic staff who enrolled and followed participants, the study team (except biostatisticians), and laboratory personnel were blinded to the randomization assignments in both trials. Site pharmacists were aware of the randomization assignments to ensure proper handling and dispensing, which included application of overlays to all syringes for blinding before delivery to clinic staff. NIAID Division of AIDS (DAIDS) protocol pharmacists, contract monitors, and data management center staff, and the NIAID data and safety monitoring board (DSMB) for HVTN 100 were unblinded to ensure proper trial conduct and safety review. Similarly for HVTN 117/HPX2004, the clinical and clinical immunology personnel involved in the analysis, and the sponsor committee involved in making future decisions for the program were unblinded. The sponsor of HVTN 117/HPX2004 was blinded to study vaccine allocation until the week 28 analysis.

## Binding antibody multiplex assay (BAMA) to measure binding antibody (bAb) response

The antigens assessed with BAMA are included in **Table B**. The readout was background-subtracted mean fluorescence intensity (MFI), where background accounts for both an antigen-specific plate level control (i.e., a blank well containing antigen-coated beads run on each plate), and a specimen-specific control (i.e., a serum well containing blank beads). The positive controls were purified polyclonal IgG from HIV-positive subjects (HIVIG) using a 10-point standard curve (4PL fit) and CH58 mAb titration. The negative controls were NHS (HIV-1 sero-negative human sera) and blank beads. The sample was repeated if the blank bead negative control exceeded 5000 MFI. If the repeat value exceeded 5000 MFI, the sample was excluded from analysis due to high background. The MFI minus blank bead responses (“net MFI”) at the specified dilutions are used to summarize the magnitude. Net MFI less than 1 was set to 1.

Samples were declared positive if the following held: (1) net MFI ≥ antigen-specific positive response threshold (defined separately for each trial as the maximum of 100 and the 95th percentile of pre-vaccination net MFI values), (2) net MFI > 3 times baseline net MFI, and (3) MFI > 3 times baseline MFI.

An individual has a positive response to a panel of antigens if they have a positive response to at least one antigen within the panel. The magnitude of responses to a panel of antigens are described by a breadth score. The breadth score is computed as the area under the Magnitude-Breadth (MB) curve for each vaccine recipient and calculated as the average of the log10(net MFI) for each antigen in the panel [1]. Net MFI values above 23,000 were truncated to 23,000 to minimize the effects of saturation in the assay readout in the calculation of the breadth score.

| **Table B.**  Antigens assessed with BAMA to determine IgG breadth of responses.  **Antigen** | **Clade** | **Country of Origin** |
| --- | --- | --- |
| **gp140 clade C Breadth Panel** |  |  |
| BF1266_gp140C.avi/293F | C | Malawi |
| C.CH505TF_gp140/293F | C |  |
| **gp140 Clade B Breadth Panel** |  |  |
| RHPA4259_C7.gp140C.avi/293F | B | USA |
| SC42261_gp140.avi/293F | B | Trinidad |
| WITO4160.gp140C.avi/293F | B | USA |
| **V1V2 Clade C Breadth Panel** |  |  |
| gp70-7060101641 V1V2 | C | South Africa |
| gp70-CAP210.2.00.E8 V1V2 | C | South Africa |
| gp70-BF1266_431a_V1V2 | C | Malawi |
| gp70-001428.2.42 V1V2 | C | India |
| gp70-BJOX002000.03.2 V1V2 | CRF07_BC | China |
| **V1V2 Clade B Breadth Panel** |  |  |
| gp70-62357.14 V1V2 | B | USA |
| gp70-700010058 V1V2 | B | USA |
| gp70-RHPA4259.7 V1V2 | B | USA |
| gp70-BCaseA V1V2 | B | USA |
| gp70-TT31P.2F10.2792 V1V2 | B | Trinidad/Tobago |
| **gp120 Clade C Breadth Panel** |  |  |
| 1394C9_G1.D11gp120.avi | C |  |
| 1428_D11gp120.avi/293F | C |  |
| 1641A7_D11gp120.avi/293F | C |  |
| CAP210_D11gp120.avi/293F | C |  |
| CAP45_D11gp120.avi/293F | C |  |
| CH505TF_D7gp120.avi/293F | C |  |
| Ce0042_D11gp120.avi/293F | C |  |
| Du156_D11gp120.avi/293F | C |  |
| **gp120 Clade B Breadth Panel** |  |  |
| B.6240_D11gp120.avi/293F | B | USA |
| BORI_D11gp120.avi/293F | B | USA |
| TT31P.2792_D11gp120.avi/293F | B | Trinidad |
| **Other Antigens** |  |  |
| gp70-BCaseA V1V2 |  |  |
| gp41 |  |  |
| Con 6 gp120/B | Consensus |  |
| Con S gp140 CFI | Consensus |  |

## Intracellular cytokine staining (ICS) to measure T-cell response

Flow cytometry was used to examine HIV-1-specific CD4+ and CD8+ T-cell responses using a validated ICS assay. A 17-color staining panel (Experiment Assay ID 109, Analysis plan 42) was used [2,3]:

| **Antibody** | **Manufacturer** | **Catalog Number** |
| --- | --- | --- |
| AViD* | Invitrogen | L34957 |
| CD3 BUV737 | BD Biosciences | 564307 |
| CD4 BUV395 | BD Biosciences | 563550 |
| CD8 BV650 | BD Biosciences | 563821 |
| CD14 BV510* | BioLegend | 301842 |
| CD56 BV570 | BioLegend | 318330 |
| CXCR5 PE-Dazzle594 | BioLegend | 356928 |
| PD-1 (CD279) BV605 | BioLegend | 329924 |
| ICOS (CD278) BV711 | BD Biosciences | 563833 |
| CD45RA APC H7 | BD Biosciences | 560674 |
| CCR7 BV786 | BioLegend | 353229 |
| IFNγ V450 | Becton Dickinson | 560371 |
| TNFα FITC | eBioscience | 11-7349-82 |
| IL2 PE | BD Biosciences | 559334 |
| IL4 PerCP-Cy5.5 | BioLegend | 500822 |
| IL17a PE-Cy7 | BioLegend | 512315 |
| CD40L APC | BD Biosciences | 555702 |
| Granzyme B Alx700 | BD Biosciences | 560213 |
| *AVID and CD14 are detected in the same channel. | | |

Previously cryopreserved PBMC specimens are stimulated *ex vivo* with the synthetic peptide pools. As a negative control, cells are not stimulated. As a positive control, cells are stimulated with a polyclonal stimulant, staphylococcal enterotoxin B (SEB). There are no replicates except for the negative control, which has two replicates.

Several criteria were used to determine if data from an assay were acceptable and could be statistically analyzed. The blood draw date must have been within the allowable visit window as determined by the protocol. Post-acquisition samples from participants who acquired HIV while on-study are excluded. After sample thawing and overnight incubation, the viability of the PBMC must have been 66% or greater for testing to have proceeded. If it was not, a new specimen for that participant at that time point was thawed for testing. If the PBMC viability of the second thawed aliquot was below this threshold, the ICS assay was not performed and no data were reported to the statistical center for the participant-time point. For the negative control acceptance criteria, if the average cytokine response for the negative control wells was above 0.1% for either the CD4+ or CD8+ T cells, the sample was retested. If the retested results were above 0.1%, the data were excluded from analysis; otherwise, the retest data were used.

The total numbers of CD4+ and CD8+ T cells must also have exceeded certain thresholds. If the number of cells was less than 5,000 CD8+ T cells or 10,000 CD4+ T cells for any of the HIV-1 peptide pools or for one of the negative control replicates for a particular sample, data for that stimulation were filtered. If both negative control replicates had low T cells, the sample was retested. If upon retesting, one negative control replicate had low T cells, the negative control replicate with enough T cells was used. If both negative control replicates from the retest for a T-cell subset had low T cells, then data for the T-cell subset were not included in the analysis.

To assess positivity for a peptide pool within a T-cell subset, a two-by-two contingency table was constructed comparing the HIV-1 peptide stimulated and negative control data. The four entries in each table were the number of cells positive for IL-2 and/or IFN-γ and the number of cells negative for IL-2 and IFN-γ, for both the stimulated and the negative control data. If both negative control replicates were included, then the average number of total cells and the average number of positive cells were used. A one-sided Fisher's exact test was applied to the table, testing whether the number of cytokine-producing cells for the stimulated data was equal to that for the negative control data. Since multiple individual tests (for each peptide pool) were conducted simultaneously, a multiplicity adjustment was made to the individual peptide pool p-values using the Bonferroni-Holm adjustment method. If the adjusted p-value for a peptide pool was ≤0.00001, the response to the peptide pool for the T-cell subset was considered positive. Because the sample sizes (i.e., total cell counts for the T-cell subset) were large, e.g., as high as 100,000 cells, the Fisher’s exact test has high power to reject the null hypothesis for very small differences. Therefore, the adjusted p-value significance threshold was chosen stringently (≤ 0.00001). If at least one peptide pool for a specific HIV-1 protein was positive, then the overall response to the protein was considered positive. If any peptide pool was positive for a T-cell subset, then the overall response for that T-cell subset was considered positive.

For these analyses, we report the pooled “Any Env” and “Any Gag” responses, defined as the maximum magnitude of the homologous peptide pools as summarized in **Table C**.

|  | **HVTN 100** | **HVTN 117** |
| --- | --- | --- |
| Any Env | 1086 gp120 (matched to the Env gp120 protein)  TV1 gp120 (matched to the Env gp120 protein)  ZM96 gp120 (matched to Env gp120 sequence in the ALVAC vector) | J Mos gp120  J Mos gp41  97ZA012 gp120  97ZA012 gp41 |
| Any Gag | LAI Gag (matched to Gag sequence in the ALVAC vector) | J Mos1 Gag |

**Table C.** Homologous peptide pools evaluated in HVTN 100 and HVTN 117 to evaluate ICS responses.

COMPASS (Combinatorial Polyfunctionality Analysis of Single Cells) is a computational framework for unbiased polyfunctionality analysis of antigen-specific T-cell subsets. COMPASS uses a Bayesian hierarchical framework to model all observed functional cell subsets and select those most likely to exhibit antigen-specific responses. Cell-subset responses are quantified by posterior probabilities, while subject-level responses are quantified by two summary statistics (“scores”) that can be correlated directly with clinical outcome and describe the quality of an individual’s (poly)functional response. The functionality score is defined as the proportion of Ag-specific subsets detected among all possible ones. The polyfunctionality score is similar, but it weighs the different subsets by their degree of functionality, naturally favoring subsets with higher degrees of functions.

ICS data included in the COMPASS analyses are restricted to samples/antigens which are deemed reliable and which visits occurred within window. Additionally, COMPASS performs two filters. Samples with T-cell subset < 5,000 are excluded. Also, cell subsets that do not have at least 5 cells in at least 2 subjects are excluded. The standard ICS filter on mean negative control is not used.

A post-hoc analysis compared response magnitudes to cell subsets identified by both protocol-specific COMPASS analyses.

## Statistical Methods

The primary analysis used super learning and targeted minimum loss-based estimation (TMLE) [4]. This method iteratively regresses the outcome on treatment assignment and the treatment assignment on covariates. The set of covariates include patient age, sex, and BMI at baseline. Each regression was estimated using the average of 10 repeated super learner runs, with 20-fold cross validation. Candidate regression estimators included in each regression are summarized in **Table D**. Although residual smoothing libraries are specified, the doubly-robust variance estimates are not used in this analysis. We report doubly-robust parameter estimates with non-doubly robust covariance estimates from the standard tmle output from the drtmle package in R.

The null hypothesis, testing that the average outcomes were the same under the two vaccine regimens used a two-sided, level 0.05 Wald test with influence function-based standard error estimates.

|  | Candidate learners included |
| --- | --- |
| Treatment on confounders (g) | c("SL.mean", "SL.glm", "SL.glm.interaction", "SL.step", "SL.step.interaction", "SL.ranger", "SL.earth", 'SL.gam') |
| Outcome on treatment (Q) | c("SL.mean", "SL.glm", "SL.glm.interaction", "SL.step", "SL.step.interaction", "SL.ranger", "SL.earth", 'SL.gam') |
| Residual smoothing (gr, Qr) | c('SL.glm', 'SL.mean', 'SL.earth', 'SL.npreg') |

**Table D.** Candidate regression estimators used in the TMLE analysis.

# Supplemental Tables

**Table E.** **Response rates (95% CIs), mean magnitudes (95% CIs), and mean magnitudes among positive responders (95% Cis) of humoral and cellular responses by unadjusted and adjusted statistical methods.**

The unadjusted estimates are based on empirical estimates from each study with nonparametric 95% CIs *where these estimates do not account for baseline covariates. The adjusted estimates are based on TMLE, accounting for age, sex assigned at birth, and BMI.*

| **Table E1. IgG gp120 clade C breadth** | | | | | | | |
| --- | --- | --- | --- | --- | --- | --- | --- |
|  |  | Unadjusted Responses | | | Adjusted Estimates from TMLE | | |
| Outcome | N | HVTN 100 | HVTN 117 | p-value | HVTN 100 | HVTN 117 | p-value |
| Month 6.5/7 | | | | | | | |
| Response rate | 41 vs 91 | 100.0% (91.43%, 100.0%) | 100.0% (95.95%, 100.0%) |  |  |  |  |
| Mean magnitude (overall) | 41 vs 91 | 4.12 (4.05, 4.18) | 3.62 (3.51, 3.72) | **<.0001** | 4.10 (4.04, 4.15) | 3.61 (3.51, 3.72) | **<.0001** |
| Mean magnitude (among positive responders) | 41 vs 91 | 4.12 (4.05, 4.18) | 3.62 (3.51, 3.72) | **<.0001** |  |  |  |
| Month 12.5/13 | | | | | | | |
| Response rate | 0 vs 83 |  | 100.0% (95.58%, 100.0%) |  |  |  |  |
| Mean magnitude (overall) | 0 vs 83 |  | 3.88 (3.79, 3.96) |  |  |  |  |
| Mean magnitude (among positive responders) | 0 vs 83 |  | 3.88 (3.79, 3.96) |  |  |  |  |
| Month 18 | | | | | | | |
| Response rate | 41 vs 82 | 87.80% (74.46%, 94.68%) | 97.56% (91.54%, 99.33%) | **0.0327** | 91.93% (84.17%, 99.68%) | 98.30% (95.71%, 100.9%) | 0.1276 |
| Mean magnitude (overall) | 41 vs 82 | 2.87 (2.69, 3.06) | 2.93 (2.77, 3.10) | 0.4179 | 2.91 (2.73, 3.1) | 2.93 (2.77, 3.09) | 0.8801 |
| Mean magnitude (among positive responders) | 36 vs 80 | 3.02 (2.88, 3.16) | 3.00 (2.87, 3.14) | 0.8720 | 3 (2.84, 3.15) | 2.97 (2.83, 3.11) | 0.8091 |

| **Table E2. IgG gp120 clade B breadth** | | | | | | | |
| --- | --- | --- | --- | --- | --- | --- | --- |
|  |  | Unadjusted Responses | | | Adjusted Estimates from TMLE | | |
| Outcome | N | HVTN 100 | HVTN 117 | p-value | HVTN 100 | HVTN 117 | p-value |
| Month 6.5/7 | | | | | | | |
| Response rate | 184 vs 91 | 100.0% (97.95%, 100.0%) | 100.0% (95.95%, 100.0%) |  |  |  |  |
| Mean magnitude (overall) | 184 vs 91 | 4.07 (4.02, 4.12) | 3.66 (3.57, 3.75) | **<.0001** | 4.07 (4.02, 4.12) | 3.65 (3.56, 3.74) | **<.0001** |
| Mean magnitude (among positive responders) | 184 vs 91 | 4.07 (4.02, 4.12) | 3.66 (3.57, 3.75) | **<.0001** |  |  |  |
| Month 12.5/13 | | | | | | | |
| Response rate | 64 vs 84 | 98.44% (91.67%, 99.72%) | 100.0% (95.63%, 100.0%) | 0.3436 |  |  |  |
| Mean magnitude (overall) | 64 vs 84 | 4.23 (4.10, 4.37) | 3.90 (3.82, 3.98) | **<.0001** | 4.16 (3.89, 4.44) | 3.89 (3.81, 3.98) | 0.0643 |
| Mean magnitude (among positive responders) | 63 vs 84 | 4.30 (4.26, 4.34) | 3.90 (3.82, 3.98) | **<.0001** | 4.31 (4.27, 4.34) | 3.88 (3.8, 3.96) | **<.0001** |
| Month 18 | | | | | | | |
| Response rate | 63 vs 83 | 95.24% (86.91%, 98.37%) | 96.39% (89.90%, 98.76%) | 0.7622 |  |  |  |
| Mean magnitude (overall) | 63 vs 83 | 3.06 (2.87, 3.26) | 2.83 (2.66, 3.00) | **0.0153** | 3.03 (2.79, 3.27) | 2.85 (2.7, 3) | 0.2032 |
| Mean magnitude (among positive responders) | 60 vs 80 | 3.17 (3.02, 3.33) | 2.88 (2.71, 3.04) | **0.0062** | 3.15 (2.95, 3.35) | 2.88 (2.73, 3.04) | **0.0340** |

| **Table E3. IgG gp140 clade C breadth** | | | | | | | |
| --- | --- | --- | --- | --- | --- | --- | --- |
|  |  | Unadjusted Responses | | | Adjusted Estimates from TMLE | | |
| Outcome | N | HVTN 100 | HVTN 117 | p-value | HVTN 100 | HVTN 117 | p-value |
| Month 6.5/7 | | | | | | | |
| Response rate | 183 vs 91 | 99.45% (96.97%, 99.90%) | 100.0% (95.95%, 100.0%) | 0.5982 |  |  |  |
| Mean magnitude (overall) | 183 vs 91 | 4.22 (4.17, 4.27) | 4.05 (3.99, 4.12) | **<.0001** | 4.23 (4.18, 4.27) | 4.06 (4, 4.12) | **<.0001** |
| Mean magnitude (among positive responders) | 182 vs 91 | 4.24 (4.22, 4.27) | 4.05 (3.99, 4.12) | **<.0001** | 4.24 (4.22, 4.27) | 4.06 (4, 4.12) | **<.0001** |
| Month 12.5/13 | | | | | | | |
| Response rate | 64 vs 84 | 98.44% (91.67%, 99.72%) | 100.0% (95.63%, 100.0%) | 0.3436 |  |  |  |
| Mean magnitude (overall) | 64 vs 84 | 4.29 (4.15, 4.42) | 4.21 (4.16, 4.26) | **<.0001** | 4.2 (3.94, 4.45) | 4.2 (4.15, 4.25) | 0.9923 |
| Mean magnitude (among positive responders) | 63 vs 84 | 4.35 (4.34, 4.37) | 4.21 (4.16, 4.26) | **<.0001** | 4.36 (4.34, 4.37) | 4.2 (4.15, 4.25) | **<.0001** |
| Month 18 | | | | | | | |
| Response rate | 63 vs 83 | 96.83% (89.14%, 99.13%) | 98.80% (93.49%, 99.79%) | 0.5641 |  |  |  |
| Mean magnitude (overall) | 63 vs 83 | 3.37 (3.16, 3.58) | 3.42 (3.29, 3.55) | 0.7988 | 3.39 (3.1, 3.67) | 3.43 (3.31, 3.54) | 0.8077 |
| Mean magnitude (among positive responders) | 61 vs 82 | 3.48 (3.33, 3.63) | 3.45 (3.34, 3.57) | 0.6330 | 3.49 (3.31, 3.67) | 3.45 (3.34, 3.55) | 0.6818 |

| **Table E4. IgG gp140 clade B breadth** | | | | | | | |
| --- | --- | --- | --- | --- | --- | --- | --- |
|  |  | Unadjusted Responses | | | Adjusted Estimates from TMLE | | |
| Outcome | N | HVTN 100 | HVTN 117 | p-value | HVTN 100 | HVTN 117 | p-value |
| Month 6.5/7 | | | | | | | |
| Response rate | 184 vs 91 | 99.46% (96.99%, 99.90%) | 100.0% (95.95%, 100.0%) | 0.5983 |  |  |  |
| Mean magnitude (overall) | 184 vs 91 | 4.03 (3.97, 4.09) | 4.09 (4.02, 4.15) | 0.0842 | 4.04 (3.99, 4.1) | 4.09 (4.03, 4.15) | 0.2132 |
| Mean magnitude (among positive responders) | 183 vs 91 | 4.05 (4.01, 4.10) | 4.09 (4.02, 4.15) | 0.0967 | 4.06 (4.01, 4.1) | 4.09 (4.03, 4.15) | 0.3789 |
| Month 12.5/13 | | | | | | | |
| Response rate | 64 vs 86 | 98.44% (91.67%, 99.72%) | 100.0% (95.72%, 100.0%) | 0.3429 |  |  |  |
| Mean magnitude (overall) | 64 vs 86 | 4.26 (4.12, 4.40) | 4.23 (4.19, 4.28) | **<.0001** | 4.18 (3.93, 4.44) | 4.23 (4.18, 4.27) | 0.7435 |
| Mean magnitude (among positive responders) | 63 vs 86 | 4.32 (4.29, 4.36) | 4.23 (4.19, 4.28) | **<.0001** | 4.33 (4.3, 4.36) | 4.23 (4.18, 4.27) | **0.0002** |
| Month 18 | | | | | | | |
| Response rate | 63 vs 84 | 93.65% (84.78%, 97.50%) | 100.0% (95.63%, 100.0%) | **0.0204** |  |  |  |
| Mean magnitude (overall) | 63 vs 84 | 2.95 (2.69, 3.21) | 3.57 (3.45, 3.69) | **<.0001** | 2.89 (2.53, 3.24) | 3.58 (3.47, 3.69) | **0.0003** |
| Mean magnitude (among positive responders) | 59 vs 84 | 3.14 (2.94, 3.34) | 3.57 (3.45, 3.69) | **0.0001** | 3.12 (2.85, 3.38) | 3.58 (3.47, 3.7) | **0.0018** |

| **Table E5. IgG V1V2 clade C breadth** | | | | | | | |
| --- | --- | --- | --- | --- | --- | --- | --- |
|  |  | Unadjusted Responses | | | Adjusted Estimates from TMLE | | |
| Outcome | N | HVTN 100 | HVTN 117 | p-value | HVTN 100 | HVTN 117 | p-value |
| Month 6.5/7 | | | | | | | |
| Response rate | 184 vs 89 | 79.89% (73.52%, 85.04%) | 86.52% (77.90%, 92.12%) | 0.1908 | 76.41% (69.72%, 83.10%) | 90.07% (84.18%, 95.96%) | **0.0026** |
| Mean magnitude (overall) | 184 vs 89 | 2.06 (1.90, 2.21) | 2.36 (2.20, 2.53) | 0.0576 | 2.01 (1.84, 2.17) | 2.38 (2.22, 2.53) | **0.0014** |
| Mean magnitude (among positive responders) | 147 vs 77 | 2.43 (2.29, 2.56) | 2.56 (2.43, 2.70) | 0.4403 | 2.47 (2.34, 2.6) | 2.57 (2.42, 2.71) | 0.3032 |
| Month 12.5/13 | | | | | | | |
| Response rate | 64 vs 84 | 95.31% (87.10%, 98.39%) | 91.67% (83.78%, 95.90%) | 0.5176 |  |  |  |
| Mean magnitude (overall) | 64 vs 84 | 3.01 (2.79, 3.22) | 2.75 (2.58, 2.91) | **0.0101** | 2.99 (2.71, 3.27) | 2.74 (2.57, 2.91) | 0.1350 |
| Mean magnitude (among positive responders) | 61 vs 77 | 3.12 (2.95, 3.29) | 2.87 (2.72, 3.02) | **0.0137** | 3.15 (2.95, 3.35) | 2.83 (2.66, 2.99) | **0.0140** |
| Month 18 | | | | | | | |
| Response rate | 63 vs 82 | 42.86% (31.40%, 55.14%) | 64.63% (53.84%, 74.11%) | **0.0095** | 40.39% (25.58%, 55.19%) | 68.00% (57.61%, 78.38%) | **0.0025** |
| Mean magnitude (overall) | 63 vs 82 | 1.00 (0.74, 1.27) | 1.75 (1.56, 1.93) | **<.0001** | 1.06 (0.69, 1.43) | 1.76 (1.58, 1.95) | **0.0008** |
| Mean magnitude (among positive responders) | 27 vs 53 | 1.92 (1.56, 2.27) | 2.17 (2.00, 2.34) | 0.1159 | 2.04 (1.51, 2.58) | 2.14 (1.96, 2.31) | 0.7472 |

| **Table E6. IgG V1V2 clade B breadth** | | | | | | | |
| --- | --- | --- | --- | --- | --- | --- | --- |
|  |  | Unadjusted Responses | | | Adjusted Estimates from TMLE | | |
| Outcome | N | HVTN 100 | HVTN 117 | p-value | HVTN 100 | HVTN 117 | p-value |
| Month 6.5/7 | | | | | | | |
| Response rate | 184 vs 89 | 62.50% (55.32%, 69.17%) | 86.52% (77.90%, 92.12%) | **<.0001** | 59.99% (52.93%, 67.05%) | 87.03% (79.63%, 94.43%) | **<.0001** |
| Mean magnitude (overall) | 184 vs 89 | 1.57 (1.41, 1.73) | 2.31 (2.12, 2.50) | **<.0001** | 1.54 (1.38, 1.7) | 2.33 (2.15, 2.51) | **<.0001** |
| Mean magnitude (among positive responders) | 115 vs 77 | 2.25 (2.11, 2.39) | 2.56 (2.41, 2.71) | **0.0011** | 2.31 (2.17, 2.45) | 2.59 (2.45, 2.73) | **0.0064** |
| Month 12.5/13 | | | | | | | |
| Response rate | 64 vs 82 | 89.06% (79.10%, 94.60%) | 90.24% (81.91%, 94.97%) | 0.8506 | 78.39% (63.26%, 93.52%) | 90.37% (83.43%, 97.32%) | 0.1580 |
| Mean magnitude (overall) | 64 vs 82 | 2.47 (2.23, 2.70) | 2.65 (2.44, 2.85) | 0.1279 | 2.37 (2.01, 2.73) | 2.64 (2.43, 2.85) | 0.2056 |
| Mean magnitude (among positive responders) | 57 vs 74 | 2.66 (2.46, 2.85) | 2.87 (2.72, 3.02) | 0.0601 | 2.68 (2.45, 2.91) | 2.87 (2.73, 3.01) | 0.1698 |
| Month 18 | | | | | | | |
| Response rate | 63 vs 81 | 25.40% (16.28%, 37.34%) | 65.43% (54.59%, 74.88%) | **<.0001** | 24.21% (12.02%, 36.39%) | 66.79% (55.92%, 77.65%) | **<.0001** |
| Mean magnitude (overall) | 63 vs 81 | 0.71 (0.49, 0.93) | 1.39 (1.18, 1.59) | **<.0001** | 0.81 (0.53, 1.09) | 1.41 (1.21, 1.6) | **0.0007** |
| Mean magnitude (among positive responders) | 16 vs 53 | 1.79 (1.33, 2.24) | 1.87 (1.67, 2.06) | 0.4860 | 1.96 (1.27, 2.65) | 1.85 (1.66, 2.03) | 0.7561 |

| **Table E7. IgG gp70-BCaseA V1V2** | | | | | | | |
| --- | --- | --- | --- | --- | --- | --- | --- |
|  |  | Unadjusted Responses | | | Adjusted Estimates from TMLE | | |
| Outcome | N | HVTN 100 | HVTN 117 | p-value | HVTN 100 | HVTN 117 | p-value |
| Month 6.5/7 | | | | | | | |
| Response rate | 184 vs 89 | 55.43% (48.21%, 62.43%) | 80.90% (71.52%, 87.72%) | **<.0001** | 53.57% (46.24%, 60.89%) | 82.41% (74.41%, 90.42%) | **<.0001** |
| Mean magnitude (overall) | 184 vs 89 | 79.98 (51.1, 125.17) | 417.12 (254.91, 682.56) | **<.0001** | 75.38 (47.14, 120.53) | 428.79 (275.76, 666.74) | **<.0001** |
| Mean magnitude (among positive responders) | 102 vs 72 | 724.4 (550.65, 952.98) | 997.79 (730.81, 1362) | 0.0821 | 818.99 (591.48, 1134) | 932.34 (684.79, 1269.4) | 0.5707 |
| Month 12.5/13 | | | | | | | |
| Response rate | 64 vs 82 | 82.81% (71.79%, 90.12%) | 90.24% (81.91%, 94.97%) | 0.2158 | 76.82% (64.87%, 88.77%) | 90.20% (83.20%, 97.21%) | 0.0573 |
| Mean magnitude (overall) | 64 vs 82 | 821.84 (439.17, 1538) | 1045 (606.64, 1799) | 0.4949 | 784.46 (375.71, 1637.92) | 983.87 (547.25, 1768.85) | 0.6369 |
| Mean magnitude (among positive responders) | 53 vs 74 | 1862 (1265, 2739) | 2021 (1457, 2803) | 0.7692 | 1915.5 (1208.74, 3035.51) | 1912.34 (1390.1, 2630.79) | 0.9954 |
| Month 18 | | | | | | | |
| Response rate | 63 vs 81 | 17.46% (10.04%, 28.62%) | 59.26% (48.38%, 69.30%) | **<.0001** | 17.75% (6.65%, 28.86%) | 61.89% (50.49%, 73.28%) | **<.0001** |
| Mean magnitude (overall) | 63 vs 81 | 5.78 (3.03, 11) | 99.89 (59.88, 166.64) | **<.0001** | 9.29 (4.43, 19.5) | 106.08 (62.95, 178.78) | **<.0001** |
| Mean magnitude (among positive responders) | 11 vs 48 | 568.05 (254.09, 1270) | 429.81 (317.35, 582.14) | 0.4246 | 767.62 (225.2, 2616.5) | 421.13 (316.42, 560.5) | 0.3492 |

| **Table E8. IgG gp41** | | | | | | | |
| --- | --- | --- | --- | --- | --- | --- | --- |
|  |  | Unadjusted Responses | | | Adjusted Estimates from TMLE | | |
| Outcome | N | HVTN 100 | HVTN 117 | p-value | HVTN 100 | HVTN 117 | p-value |
| Month 6.5/7 | | | | | | | |
| Response rate | 184 vs 91 | 0.54% ( 0.10%, 3.01%) | 100.0% (95.95%, 100.0%) | **<.0001** |  |  |  |
| Mean magnitude (overall) | 184 vs 91 | 178.81 (132.52, 241.26) | 23000 (23000, 23000) | **<.0001** |  |  |  |
| Mean magnitude (among positive responders) | 1 vs 91 | 6636 | 23000 (23000, 23000) | **<.0001** |  |  |  |
| Month 12.5/13 | | | | | | | |
| Response rate | 64 vs 86 | 1.56% ( 0.28%, 8.33%) | 100.0% (95.72%, 100.0%) | **<.0001** |  |  |  |
| Mean magnitude (overall) | 64 vs 86 | 314.54 (182.39, 542.42) | 23000 (23000, 23000) | **<.0001** |  |  |  |
| Mean magnitude (among positive responders) | 1 vs 86 | 5598 | 23000 (23000, 23000) | **<.0001** |  |  |  |
| Month 18 | | | | | | | |
| Response rate | 63 vs 84 | 4.76% ( 1.63%, 13.09%) | 100.0% (95.63%, 100.0%) | **<.0001** |  |  |  |
| Mean magnitude (overall) | 63 vs 84 | 285.24 (159.09, 511.42) | 20816 (19563, 22151) | **<.0001** |  |  |  |
| Mean magnitude (among positive responders) | 3 vs 84 | 5544 (841.88, 36504) | 20816 (19563, 22151) | **<.0001** |  |  |  |

| **Table E9. IgG3 Con 6 gp120/B** | | | | | | | |
| --- | --- | --- | --- | --- | --- | --- | --- |
|  |  | Unadjusted Responses | | | Adjusted Estimates from TMLE | | |
| Outcome | N | HVTN 100 | HVTN 117 | p-value | HVTN 100 | HVTN 117 | p-value |
| Month 6.5/7 | | | | | | | |
| Response rate | 183 vs 98 | 63.39% (56.20%, 70.03%) | 51.02% (41.27%, 60.69%) | **0.0476** | 62.02% (54.77%, 69.28%) | 53.64% (42.80%, 64.48%) | 0.2090 |
| Mean magnitude (overall) | 183 vs 98 | 137.62 (110.41, 171.53) | 104.39 (69.28, 157.29) | 0.2592 | 126.09 (99.36, 160.01) | 117.37 (83.47, 165.05) | 0.7363 |
| Mean magnitude (among positive responders) | 116 vs 50 | 311.82 (266.04, 365.46) | 479.43 (353.55, 650.13) | **0.0154** | 317.26 (272.2, 369.78) | 425.65 (276.77, 654.62) | 0.2042 |
| Month 12.5/13 | | | | | | | |
| Response rate | 64 vs 93 | 25.00% (16.01%, 36.82%) | 46.24% (36.45%, 56.32%) | **0.0071** | 20.59% (11.25%, 29.94%) | 47.02% (36.29%, 57.76%) | **0.0002** |
| Mean magnitude (overall) | 64 vs 93 | 26.37 (17.46, 39.82) | 104.06 (68.79, 157.41) | **<.0001** | 23.93 (16.39, 34.94) | 107.33 (71.69, 160.68) | **<.0001** |
| Mean magnitude (among positive responders) | 16 vs 43 | 188.88 (145.07, 245.92) | 511.73 (360.76, 725.87) | **0.0006** | 173.67 (130.58, 230.97) | 490.49 (356.5, 674.86) | **<.0001** |
| Month 18 | | | | | | | |
| Response rate | 63 vs 91 | 1.59% ( 0.28%, 8.46%) | 14.29% ( 8.54%, 22.92%) | **0.0073** | 1.05% (-1.08%, 3.18%) | 13.75% (6.23%, 21.28%) | **0.0015** |
| Mean magnitude (overall) | 63 vs 91 | 2.43 (1.81, 3.26) | 16.01 (10.69, 23.97) | **<.0001** | 2.13 (1.68, 2.69) | 16.87 (11.31, 25.17) | **<.0001** |
| Mean magnitude (among positive responders) | 1 vs 13 | 145 | 300.66 (175, 516.55) | 0.7143 |  |  |  |

| **Table E10. IgG3 Con S gp140 CFI** | | | | | | | |
| --- | --- | --- | --- | --- | --- | --- | --- |
|  |  | Unadjusted Responses | | | Adjusted Estimates from TMLE | | |
| Outcome | N | HVTN 100 | HVTN 117 | p-value | HVTN 100 | HVTN 117 | p-value |
| Month 6.5/7 | | | | | | | |
| Response rate | 183 vs 98 | 81.42% (75.16%, 86.39%) | 92.86% (85.98%, 96.50%) | **0.0099** | 80.77% (74.91%, 86.62%) | 95.01% (91.18%, 98.83%) | **<.0001** |
| Mean magnitude (overall) | 183 vs 98 | 312.23 (246.18, 395.99) | 1064 (758.99, 1490) | **<.0001** | 296.34 (234.43, 374.6) | 1133.88 (844.08, 1523.19) | **<.0001** |
| Mean magnitude (among positive responders) | 149 vs 91 | 520.09 (434.73, 622.21) | 1383 (1040, 1840) | **<.0001** | 504.27 (416.66, 610.3) | 1308.04 (989.59, 1728.96) | **<.0001** |
| Month 12.5/13 | | | | | | | |
| Response rate | 64 vs 93 | 50.00% (38.10%, 61.90%) | 89.25% (81.33%, 94.05%) | **<.0001** | 42.10% (29.16%, 55.05%) | 89.12% (82.33%, 95.90%) | **<.0001** |
| Mean magnitude (overall) | 64 vs 93 | 101.32 (63.31, 162.17) | 906.11 (604.08, 1359) | **<.0001** | 91.44 (59.35, 140.89) | 859.59 (566.26, 1304.89) | **<.0001** |
| Mean magnitude (among positive responders) | 32 vs 83 | 383.47 (268.94, 546.78) | 1410 (1038, 1916) | **<.0001** | 343.22 (236.02, 499.12) | 1386.39 (1033.72, 1859.37) | **<.0001** |
| Month 18 | | | | | | | |
| Response rate | 63 vs 91 | 6.35% ( 2.50%, 15.22%) | 65.93% (55.73%, 74.85%) | **<.0001** | 5.51% (0.00%, 11.02%) | 66.46% (56.01%, 76.91%) | **<.0001** |
| Mean magnitude (overall) | 63 vs 91 | 7.51 (4.82, 11.69) | 181.01 (120.26, 272.46) | **<.0001** | 8.12 (5.02, 13.16) | 186.12 (128.47, 269.62) | **<.0001** |
| Mean magnitude (among positive responders) | 4 vs 60 | 330.78 (40.93, 2674) | 535.44 (402.01, 713.15) | 0.3601 | 818.47 (35.08, 19097.63) | 535.88 (404.35, 710.19) | 0.7927 |

| **Table E11. IgG3 gp70 BCaseA V1V2** | | | | | | | |
| --- | --- | --- | --- | --- | --- | --- | --- |
|  |  | Unadjusted Responses | | | Adjusted Estimates from TMLE | | |
| Outcome | N | HVTN 100 | HVTN 117 | p-value | HVTN 100 | HVTN 117 | p-value |
| Month 6.5/7 | | | | | | | |
| Response rate | 182 vs 96 | 8.24% ( 5.06%, 13.15%) | 13.54% ( 8.09%, 21.80%) | 0.1714 | 6.71% (3.35%, 10.08%) | 14.67% (6.08%, 23.26%) | 0.0907 |
| Mean magnitude (overall) | 182 vs 96 | 2.72 (2.05, 3.61) | 6.14 (3.98, 9.48) | **0.0002** | 2.64 (2.03, 3.44) | 6.36 (3.82, 10.61) | **0.0028** |
| Mean magnitude (among positive responders) | 15 vs 13 | 439.51 (252.33, 765.52) | 279.38 (176.72, 441.67) | 0.2005 | 417.75 (199.62, 874.26) | 285.42 (147.98, 550.54) | 0.4361 |
| Month 12.5/13 | | | | | | | |
| Response rate | 63 vs 91 | 9.52% ( 4.44%, 19.26%) | 10.99% ( 6.08%, 19.06%) | 0.7945 | 9.57% (1.41%, 17.73%) | 10.31% (3.65%, 16.97%) | 0.8900 |
| Mean magnitude (overall) | 63 vs 91 | 3.24 (1.92, 5.47) | 5.56 (3.59, 8.61) | 0.0580 | 3.6 (1.91, 6.75) | 5.3 (3.42, 8.24) | 0.3218 |
| Mean magnitude (among positive responders) | 6 vs 10 | 395.76 (130.48, 1200) | 319.2 (149.18, 682.99) | 0.7128 | 282.03 (74.06, 1073.95) | 320.72 (132.24, 777.81) | 0.8768 |
| Month 18 | | | | | | | |
| Response rate | 63 vs 91 | 3.17% (0.87%, 10.86%) | 1.10% (0.19%, 5.96%) | 0.4278 |  |  |  |
| Mean magnitude (overall) | 63 vs 91 | 1.24 (0.99, 1.56) | 1.69 (1.3, 2.2) | **0.0338** | 1.4 (1, 1.96) | 1.63 (1.26, 2.1) | 0.4792 |
| Mean magnitude (among positive responders) | 2 vs 1 | 117.5 (113.75, 121.37) | 653 | 0.6667 |  |  |  |

| **Table E12. IgG3 gp41** | | | | | | | |
| --- | --- | --- | --- | --- | --- | --- | --- |
|  |  | Unadjusted Responses | | | Adjusted Estimates from TMLE | | |
| Outcome | N | HVTN 100 | HVTN 117 | p-value | HVTN 100 | HVTN 117 | p-value |
| Month 6.5/7 | | | | | | | |
| Response rate | 183 vs 98 | 0.00% (0.00%, 2.06%) | 96.94% (91.38%, 98.95%) | **<.0001** |  |  |  |
| Mean magnitude (overall) | 183 vs 98 | 45.19 (35.75, 57.12) | 6751 (4921, 9261) | **<.0001** |  |  |  |
| Mean magnitude (among positive responders) | 0 vs 95 |  | 8044 (6332, 10217) |  |  |  |  |
| Month 12.5/13 | | | | | | | |
| Response rate | 64 vs 93 | 0.00% (0.00%, 5.66%) | 96.77% (90.94%, 98.90%) | **<.0001** |  |  |  |
| Mean magnitude (overall) | 64 vs 93 | 30.81 (21.37, 44.42) | 6011 (4314, 8376) | **<.0001** |  |  |  |
| Mean magnitude (among positive responders) | 0 vs 90 |  | 7186 (5564, 9281) |  |  |  |  |
| Month 18 | | | | | | | |
| Response rate | 63 vs 91 | 0.00% (0.00%, 5.75%) | 84.62% (75.82%, 90.61%) | **<.0001** |  |  |  |
| Mean magnitude (overall) | 63 vs 91 | 46.05 (32.29, 65.68) | 1632 (1134, 2350) | **<.0001** |  |  |  |
| Mean magnitude (among positive responders) | 0 vs 77 |  | 2483 (1754, 3515) |  |  |  |  |

| **Table E13. CD4+ Env** | | | | | | | |
| --- | --- | --- | --- | --- | --- | --- | --- |
|  |  | Unadjusted Responses | | | Adjusted Estimates from TMLE | | |
| Outcome | N | HVTN 100 | HVTN 117 | p-value | HVTN 100 | HVTN 117 | p-value |
| Month 6.5/7 | | | | | | | |
| Response rate | 67 vs 97 | 70.15% ( 58.34% , 79.77% ) | 56.70% ( 46.77% , 66.12% ) | **0.0137** | 61.65% (48.09%, 75.21%) | 56.10% (45.17%, 67.03%) | 0.5337 |
| Mean magnitude (overall) | 67 vs 97 | 0.1243 (0.1047, 0.1475) | 0.0999 (0.0874, 0.1141) | 0.2011 | 0.0995 (0.0755, 0.1311) | 0.0988 (0.0812, 0.1202) | 0.9674 |
| Mean magnitude (among positive responders) | 47 vs 55 | 0.1991 (0.1692, 0.2344) | 0.1862 (0.1631, 0.2127) | 0.9598 | 0.1967 (0.1551, 0.2495) | 0.1802 (0.1447, 0.2246) | 0.6011 |
| Month 12.5/13 | | | | | | | |
| Response rate | 66 vs 91 | 80.30% (69.16%, 88.11%) | 75.82% (66.10%, 83.46%) | 0.3614 | 78.15% (65.75%, 90.55%) | 76.06% (66.73%, 85.40%) | 0.7921 |
| Mean magnitude (overall) | 66 vs 91 | 0.1466 (0.1264, 0.1700) | 0.1212 (0.1075, 0.1365) | 0.3931 | 0.1362 (0.1057, 0.1756) | 0.1211 (0.1015, 0.1444) | 0.4494 |
| Mean magnitude (among positive responders) | 53 vs 69 | 0.1899 (0.1646, 0.2191) | 0.1624 (0.1457, 0.1809) | 0.2909 | 0.1765 (0.1413, 0.2205) | 0.1607 (0.1381, 0.187) | 0.4922 |
| Month 18 | | | | | | | |
| Response rate | 64 vs 78 | 53.13% ( 41.07% , 64.82% ) | 64.10% ( 53.03% , 73.85% ) | 0.0668 | 48.91% (34.64%, 63.17%) | 65.43% (54.09%, 76.77%) | 0.0732 |
| Mean magnitude (overall) | 64 vs 78 | 0.0785 (0.0667, 0.0924) | 0.0956 (0.0838, 0.1091) | **0.0499** | 0.0758 (0.0565, 0.1017) | 0.0974 (0.0797, 0.1191) | 0.1607 |
| Mean magnitude (among positive responders) | 34 vs 50 | 0.1531 (0.1279, 0.1832) | 0.1534 (0.1363, 0.1728) | 0.8625 | 0.1654 (0.1192, 0.2294) | 0.1533 (0.129, 0.1822) | 0.6846 |

| **Table E14. CD4+ Gag** | | | | | | | |
| --- | --- | --- | --- | --- | --- | --- | --- |
|  |  | Unadjusted Responses | | | Adjusted Estimates from TMLE | | |
| Outcome | N | HVTN 100 | HVTN 117 | p-value | HVTN 100 | HVTN 117 | p-value |
| Month 6.5/7 | | | | | | | |
| Response rate | 67 vs 97 | 5.97% ( 2.35%, 14.37%) | 8.25% ( 4.24%, 15.44%) | 0.5719 |  |  |  |
| Mean magnitude (overall) | 67 vs 97 | 0.0304 (0.0269, 0.0343) | 0.0295 (0.0282, 0.0309) | 0.0817 | 0.0292 (0.0254, 0.0334) | 0.0295 (0.0275, 0.0317) | 0.8668 |
| Mean magnitude (among positive responders) | 4 vs 8 | 0.3060 (0.0763, 1.2268) | 0.0681 (0.0596, 0.0779) | 0.2141 |  |  |  |
| Month 12.5/13 | | | | | | | |
| Response rate | 66 vs 91 | 7.58% ( 3.28%, 16.54%) | 3.30% ( 1.13%, 9.25%) | 0.1011 |  |  |  |
| Mean magnitude (overall) | 66 vs 91 | 0.0304 (0.0268, 0.0344) | 0.0276 (0.0266, 0.0287) | 0.1888 | 0.0296 (0.0253, 0.0347) | 0.0274 (0.026, 0.0289) | 0.3652 |
| Mean magnitude (among positive responders) | 5 vs 3 | 0.2097 (0.0620, 0.7085) | 0.0765 (0.0507, 0.1155) | 0.5714 |  |  |  |
| Month 18 | | | | | | | |
| Response rate | 64 vs 78 | 4.69% ( 1.61%, 12.90%) | 2.56% ( 0.71%, 8.88%) | 0.3682 |  |  |  |
| Mean magnitude (overall) | 64 vs 78 | 0.0283 (0.0255, 0.0314) | 0.0276 (0.0267, 0.0285) | 0.1631 | 0.028 (0.0247, 0.0317) | 0.0282 (0.0266, 0.0298) | 0.9110 |
| Mean magnitude (among positive responders) | 3 vs 2 | 0.2874 (0.0607, 1.3603) | 0.0493 (0.0434, 0.0560) | 0.2000 |  |  |  |

| **Table E15. CD8+ Env** | | | | | | | |
| --- | --- | --- | --- | --- | --- | --- | --- |
|  |  | Unadjusted Responses | | | Adjusted Estimates from TMLE | | |
| Outcome | N | HVTN 100 | HVTN 117 | p-value | HVTN 100 | HVTN 117 | p-value |
| Month 6.5/7 | | | | | | | |
| Response rate | 70 vs 96 | 0.00% ( 0.00%, 5.20%) | 41.67% (32.31%, 51.66%) | **<.0001** |  |  |  |
| Mean magnitude (overall) | 70 vs 96 | 0.0254 (0.0249, 0.0259) | 0.0829 (0.0692, 0.0994) | **<.0001** |  |  |  |
| Mean magnitude (among positive responders) | 0 vs 40 |  | 0.2846 (0.2292, 0.3534) |  |  |  |  |
| Month 12.5/13 | | | | | | | |
| Response rate | 68 vs 91 | 1.47% ( 0.26%, 7.87%) | 34.07% (25.15%, 44.27%) | **<.0001** |  |  |  |
| Mean magnitude (overall) | 68 vs 91 | 0.0263 (0.0252, 0.0273) | 0.0646 (0.0545, 0.0764) | **<.0001** |  |  |  |
| Mean magnitude (among positive responders) | 1 vs 31 | 0.0443 (0.0443, 0.0443) | 0.2527 (0.2025, 0.3154) | 0.0625 |  |  |  |
| Month 18 | | | | | | | |
| Response rate | 65 vs 79 | 1.54% ( 0.27%, 8.21%) | 34.18% (24.67%, 45.15%) | **<.0001** |  |  |  |
| Mean magnitude (overall) | 65 vs 79 | 0.0259 (0.0251, 0.0267) | 0.0630 (0.0527, 0.0753) | **<.0001** |  |  |  |
| Mean magnitude (among positive responders) | 1 vs 27 | 0.0783 (0.0783, 0.0783) | 0.2230 (0.1718, 0.2894) | 0.3571 |  |  |  |

| **Table E16. CD8+ Gag** | | | | | | | |
| --- | --- | --- | --- | --- | --- | --- | --- |
|  |  | Unadjusted Responses | | | Adjusted Estimates from TMLE | | |
| Outcome | N | HVTN 100 | HVTN 117 | p-value | HVTN 100 | HVTN 117 | p-value |
| Month 6.5/7 | | | | | | | |
| Response rate | 70 vs 96 | 0.00% ( 0.00%, 5.20%) | 39.58% (30.38%, 49.58%) | **<.0001** |  |  |  |
| Mean magnitude (overall) | 70 vs 96 | 0.0251 (0.0250, 0.0251) | 0.0816 (0.0658, 0.1013) | **<.0001** |  |  |  |
| Mean magnitude (among positive responders) | 0 vs 38 |  | 0.4301 (0.3369, 0.5491) |  |  |  |  |
| Month 12.5/13 | | | | | | | |
| Response rate | 68 vs 91 | 0.00% ( 0.00%, 5.35%) | 31.87% (23.20%, 42.01%) | **<.0001** |  |  |  |
| Mean magnitude (overall) | 68 vs 91 | 0.0250 (0.0250, 0.0250) | 0.0666 (0.0543, 0.0817) | **<.0001** |  |  |  |
| Mean magnitude (among positive responders) | 0 vs 29 |  | 0.4074 (0.3096, 0.5361) |  |  |  |  |
| Month 18 | | | | | | | |
| Response rate | 65 vs 79 | 0.00% ( 0.00%, 5.58%) | 32.91% (23.55%, 43.85%) | **<.0001** |  |  |  |
| Mean magnitude (overall) | 65 vs 79 | 0.0250 (0.0250, 0.0250) | 0.0650 (0.0529, 0.0799) | **<.0001** |  |  |  |
| Mean magnitude (among positive responders) | 0 vs 26 |  | 0.3311 (0.2486, 0.4410) |  |  |  |  |

| **Table E17. Overall magnitude of CD4+ and CD8+ polyfunctionality scores (PFS) to Env and Gag** | | | | | | | |
| --- | --- | --- | --- | --- | --- | --- | --- |
|  |  | Unadjusted Responses | | | Adjusted Estimates from TMLE | | |
| Timepoint | N | HVTN 100 | HVTN 117 | p-value | HVTN 100 | HVTN 117 | p-value |
| CD4+ Env | | | | | | | |
| Month 6.5/7 | 70 vs 97 | 0.0345  (0.0323, 0.0368) | 0.0224  (0.0212, 0.0237) | **<.0001** | 0.0328  (0.0308, 0.035) | 0.0227  (0.0216, 0.0239) | **<.0001** |
| Month 12.5/13 | 68 vs 92 | 0.0365  (0.0345, 0.0386) | 0.0235  (0.0220, 0.0251) | **<.0001** | 0.0333  (0.0313, 0.0354) | 0.0232  (0.0215, 0.0251) | **<.0001** |
| Month 18 | 66 vs 74 | 0.0290  (0.0270, 0.0311) | 0.0207  (0.0193, 0.0222) | **<.0001** | 0.0279  (0.0263, 0.0296) | 0.0206  (0.0191, 0.0222) | **<.0001** |
| CD4+ Gag | | | | | | | |
| Month 6.5/7 | 70 vs 97 | 0.0009  (0.0005, 0.0016) | 0.0069  (0.0060, 0.0079) | **<.0001** | 0.001  (0.0007, 0.0016) | 0.0071  (0.0062, 0.0082) | **<.0001** |
| Month 12.5/13 | 68 vs 92 | 0.0010  (0.0006, 0.0018) | 0.0059  (0.0048, 0.0071) | **<.0001** | 0.0011  (0.0007, 0.0017) | 0.0059  (0.0048, 0.0072) | **<.0001** |
| Month 18 | 66 vs 74 | 0.0010  (0.0007, 0.0015) | 0.0040  (0.0026, 0.0061) | **<.0001** | 0.001  (0.0007, 0.0014) | 0.0044  (0.0029, 0.0068) | **0.0007** |
| CD8+ Env | | | | | | | |
| Month 6.5/7 | 70 vs 97 | 0.0050  (0.0044, 0.0058) | 0.0138  (0.0122, 0.0155) | **<.0001** | 0.0046  (0.0037, 0.0055) | 0.0144  (0.0128, 0.0161) | **<.0001** |
| Month 12.5/13 | 68 vs 92 | 0.0042  (0.0031, 0.0057) | 0.0131  (0.0114, 0.0151) | **<.0001** | 0.0043  (0.0034, 0.0053) | 0.0137  (0.0119, 0.0157) | **<.0001** |
| Month 18 | 66 vs 74 | 0.0037  (0.0025, 0.0055) | 0.0111  (0.0095, 0.0130) | **<.0001** | 0.0043  (0.0034, 0.0055) | 0.012  (0.0101, 0.0143) | **<.0001** |
| CD8+ Gag | | | | | | | |
| Month 6.5/7 | 70 vs 97 | 0.0000  (0.0000, 0.0001) | 0.0093  (0.0068, 0.0126) | **<.0001** | 0  (0, 0.0001) | 0.0095  (0.0069, 0.0132) | **<.0001** |
| Month 12.5/13 | 68 vs 92 | 0.0001  (0.0000, 0.0002) | 0.0094  (0.0069, 0.0127) | **<.0001** | 0.0001  (0, 0.0001) | 0.0106  (0.0083, 0.0137) | **<.0001** |
| Month 18 | 66 vs 74 | 0.0000  (0.0000, 0.0000) | 0.0077  (0.0053, 0.0110) | **<.0001** | 0  (0, 0.0001) | 0.0077  (0.0049, 0.0121) | **<.0001** |

| **Table E18. Overall magnitude of CD4+ and CD8+ functionality scores (FS) to Env and Gag** | | | | | | | |
| --- | --- | --- | --- | --- | --- | --- | --- |
|  |  | Unadjusted Responses | | | Adjusted Estimates from TMLE | | |
| Timepoint | N | HVTN 100 | HVTN 117 | p-value | HVTN 100 | HVTN 117 | p-value |
| CD4+ Env | | | | | | | |
| Month 6.5/7 | 70 vs 97 | 0.0709  (0.0665, 0.0756) | 0.0493  (0.0466, 0.0521) | **<.0001** |  |  |  |
| Month 12.5/13 | 68 vs 92 | 0.0759  (0.0721, 0.0799) | 0.0516  (0.0485, 0.0549) | **<.0001** |  |  |  |
| Month 18 | 66 vs 74 | 0.0602  (0.0563, 0.0644) | 0.0459  (0.0429, 0.0492) | **<.0001** |  |  |  |
| CD4+ Gag | | | | | | | |
| Month 6.5/7 | 70 vs 97 | 0.0019  (0.001, 0.0033) | 0.0151  (0.0132, 0.0172) | **<.0001** |  |  |  |
| Month 12.5/13 | 68 vs 92 | 0.002  (0.0011, 0.0035) | 0.0129  (0.0107, 0.0154) | **<.0001** |  |  |  |
| Month 18 | 66 vs 74 | 0.0021  (0.0014, 0.003) | 0.0083  (0.0052, 0.0132) | **<.0001** |  |  |  |
| CD8+ Env | | | | | | | |
| Month 6.5/7 | 70 vs 97 | 0.008  (0.007, 0.0092) | 0.0278  (0.0245, 0.0315) | **<.0001** |  |  |  |
| Month 12.5/13 | 68 vs 92 | 0.0067  (0.005, 0.0092) | 0.0261  (0.0225, 0.0302) | **<.0001** |  |  |  |
| Month 18 | 66 vs 74 | 0.0059  (0.004, 0.0089) | 0.0228  (0.0195, 0.0267) | **<.0001** |  |  |  |
| CD8+ Gag | | | | | | | |
| Month 6.5/7 | 70 vs 97 | 0 (0, 0) | 0.0191  (0.0139, 0.0263) | **<.0001** |  |  |  |
| Month 12.5/13 | 68 vs 92 | 0 (0, 0) | 0.019  (0.0138, 0.0261) | **<.0001** |  |  |  |
| Month 18 | 66 vs 74 | 0 (0, 0) | 0.0159  (0.011, 0.0231) | **<.0001** |  |  |  |

**Table F. Mean magnitudes (95% CIs) of CD4+ T-cell responses to COMPASS identified, Env-specific marker subsets at Month 12.5/13 by unadjusted and adjusted statistical methods.**

The unadjusted estimates are based on empirical estimates from each study with nonparametric 95% CIs where these estimates do not account for baseline covariates. The adjusted estimates are based on TMLE, accounting for age, sex assigned at birth, and BMI. Empty cells in unadjusted column indicate that the subset was filtered by the protocol-specific COMPASS analysis due to low posterior probabilities.

| **CD4+ Env COMPASS-identified marker subsets and Month 12.5/13** | | | | | | |
| --- | --- | --- | --- | --- | --- | --- |
|  | Unadjusted Geometric Mean | | | Adjusted Estimates from TMLE | | |
| Cellular Subset | HVTN 100 | HVTN 117 | p-value | HVTN 100 | HVTN 117 | p-value |
| IFNγ | 0.003  (0.000, 0.021) | 0.001  (0.000, 0.004) | **<0.001** | 0.003  (0.002, 0.004) | 0.001  (0.001, 0.001) | **<0.001** |
| IL2 | 0.002  (0.000, 0.010) | 0.001  (0.000, 0.003) | **<0.001** | 0.002  (0.001, 0.002) | 0.001  (0.001, 0.001) | **0.004** |
| TNFα | 0.002  (0.000, 0.013) | 0.001  (0.000, 0.003) | 0.822 | 0.002  (0.001, 0.002) | 0.001  (0.001, 0.001) | 0.078 |
| CD40L | 0.044  (0.007, 0.288) | 0.026  (0.002, 0.453) | **0.026** | 0.036  (0.025, 0.052) | 0.028  (0.020, 0.037) | 0.267 |
| IL4 | 0.001  (0.001, 0.003) |  |  |  |  |  |
| IL17a | 0.002  (0.000, 0.006) |  |  |  |  |  |
| IFNγ, TNFα | 0.002  (0.000, 0.015) |  |  |  |  |  |
| IL2, TNFα | 0.002  (0.000, 0.017) | 0.001  (0.000, 0.005) | **0.002** | 0.002  (0.002, 0.003) | 0.001  (0.001, 0.002) | **0.009** |
| IFNγ, CD40L | 0.006  (0.001, 0.054) | 0.006  (0.001, 0.044) | 0.982 | 0.006  (0.004, 0.008) | 0.006  (0.005, 0.008) | 0.594 |
| IL2, CD40L | 0.015  (0.003, 0.082) | 0.011  (0.001, 0.079) | 0.099 | 0.015  (0.013, 0.019) | 0.010  (0.008, 0.013) | **0.005** |
| TNFα, CD40L | 0.029  (0.006, 0.136) | 0.008  (0.001, 0.070) | **<0.001** | 0.028  (0.024, 0.033) | 0.008  (0.006, 0.010) | **<0.001** |
| TNFα, IL17a | 0.002  (0.000, 0.008) |  |  |  |  |  |
| IL4, GzB | 0.001  (0.001, 0.002) |  |  |  |  |  |
| IFNγ, IL2, TNFα | 0.002  (0.000, 0.005) |  |  |  |  |  |
| IFNγ, IL2, CD40L | 0.002  (0.000, 0.013) | 0.005  (0.001, 0.036) | **<0.001** | 0.002  (0.002, 0.003) | 0.005  (0.004, 0.006) | **<0.001** |
| IFNγ, TNFα, CD40L | 0.008  (0.001, 0.079) | 0.006  (0.001, 0.042) | 0.113 | 0.007  (0.005, 0.010) | 0.006  (0.005, 0.008) | 0.566 |
| IL2, TNFα, CD40L | 0.040  (0.004, 0.391) | 0.014  (0.001, 0.128) | **<0.001** | 0.040  (0.031, 0.051) | 0.013  (0.010, 0.017) | **<0.001** |
| IL2, TNFα, IL17a | 0.001  (0.001, 0.002) |  |  |  |  |  |
| TNFα, CD40L, IL17a | 0.002  (0.000, 0.013) |  |  |  |  |  |
| IFNγ, IL2, TNFα, CD40L | 0.011  (0.001, 0.175) | 0.016  (0.002, 0.140) | 0.119 | 0.010  (0.007, 0.014) | 0.016  (0.013, 0.020) | **0.028** |
| IL2, TNFα, CD40L, IL4 | 0.003  (0.000, 0.033) |  |  |  |  |  |
| IL2, TNFα, CD40L, IL17a | 0.002  (0.000, 0.008) |  |  |  |  |  |
| IFNγ, IL2, TNFα, CD40L, IL4 | 0.001  (0.000, 0.004) |  |  |  |  |  |

**Table G. Mean magnitudes (95% CIs) of CD8+ T-cell responses to COMPASS identified, Env-specific marker subsets at Month 12.5/13 by unadjusted and adjusted statistical methods.**

The *unadjusted estimates are based on empirical estimates from each study with nonparametric 95% CIs where these estimates do not account for baseline covariates. The adjusted estimates are based on TMLE, accounting for age, sex assigned at birth, and BMI.*

| **CD8+ Env COMPASS-identified marker subsets and Month 12.5/13** | | | | | | |
| --- | --- | --- | --- | --- | --- | --- |
|  | Unadjusted Geometric Mean | | | Adjusted Estimates from TMLE | | |
| Cellular Subset | HVTN 100 | HVTN 117 | p-value | HVTN 100 | HVTN 117 | p-value |
| IFNγ | 0.001  (0.000, 0.006) | 0.004  (0.000, 0.038) | **<0.001** | 0.002  (0.001, 0.003) | 0.004  (0.003, 0.005) | **<0.001** |
| TNFα | 0.002  (0.000, 0.008) | 0.002  (0.000, 0.023) | **0.009** | 0.002  (0.001, 0.002) | 0.003  (0.002, 0.004) | 0.061 |
| CD40L | 0.002  (0.000, 0.007) | 0.001  (0.000, 0.006) | 0.167 |  |  |  |
| IL4 | 0.001  (0.001, 0.001) |  |  |  |  |  |
| IFNγ, TNFα | 0.001  (0.000, 0.007) | 0.008  (0.000, 0.240) | **<0.001** | 0.002  (0.001, 0.002) | 0.009  (0.006, 0.013) | **<0.001** |
| IFNγ, GzB | 0.001  (0.000, 0.006) | 0.005  (0.000, 0.083) | **<0.001** | 0.001  (0.001, 0.002) | 0.006  (0.004, 0.008) | **<0.001** |
| TNFα, GzB | 0.002  (0.000, 0.010) | 0.004  (0.000, 0.065) | **<0.001** | 0.002  (0.001, 0.002) | 0.004  (0.003, 0.006) | **<0.001** |
| CD40L, GzB | 0.002  (0.000, 0.009) | 0.002  (0.000, 0.007) | 0.611 | 0.002  (0.001, 0.002) | 0.002  (0.001, 0.002) | 0.684 |
| IL4, GzB | 0.001  (0.001, 0.001) |  |  |  |  |  |
| IFNγ, IL2, TNFα |  | 0.002  (0.000, 0.023) |  |  |  |  |
| IFNγ, TNFα, GzB | 0.002  (0.000, 0.011) | 0.014  (0.000, 0.553) | **<0.001** | 0.002  (0.001, 0.003) | 0.016  (0.010, 0.024) | **<0.001** |
| IFNγ, IL2, TNFα, GzB |  | 0.002  (0.000, 0.013) |  |  |  |  |
| IFNγ, TNFα, CD40L, GzB |  | 0.001  (0.001, 0.001) |  |  |  |  |

# Supplemental Figures


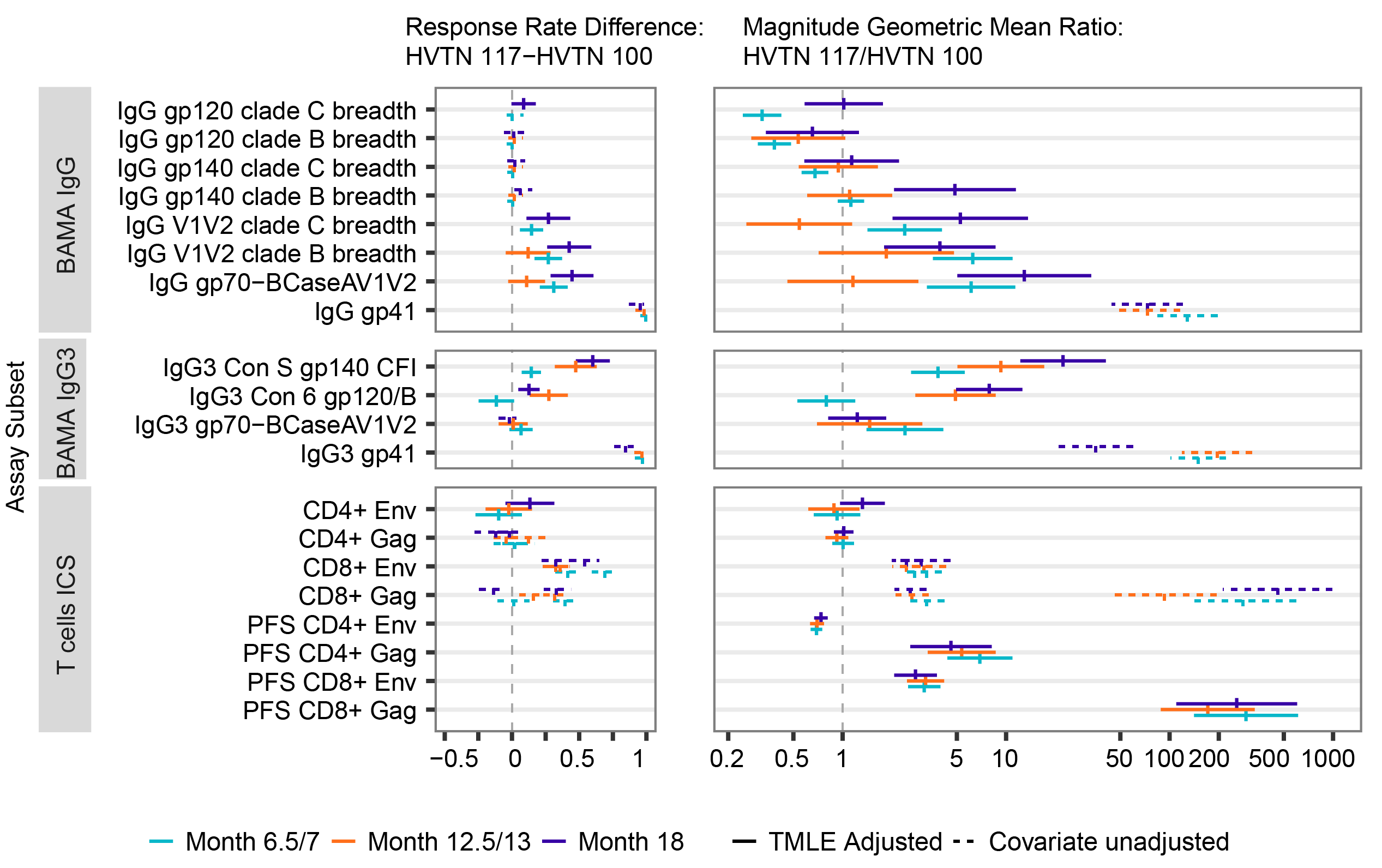


**Fig A. Forest plot summarizing the differences in immune responses at Month 6.5 (light blue), Month 12.5 (orange), and Month 18 (purple).**

Risk differences (95% CIs) and geometric mean ratios (95% CIs) with higher responses in HVTN 117/HPX2004 than in HVTN 100 are on the right of the vertical dashed lines. Covariate unadjusted results are presented only when TMLE analyses were not possible (due to very high or very low response rates).


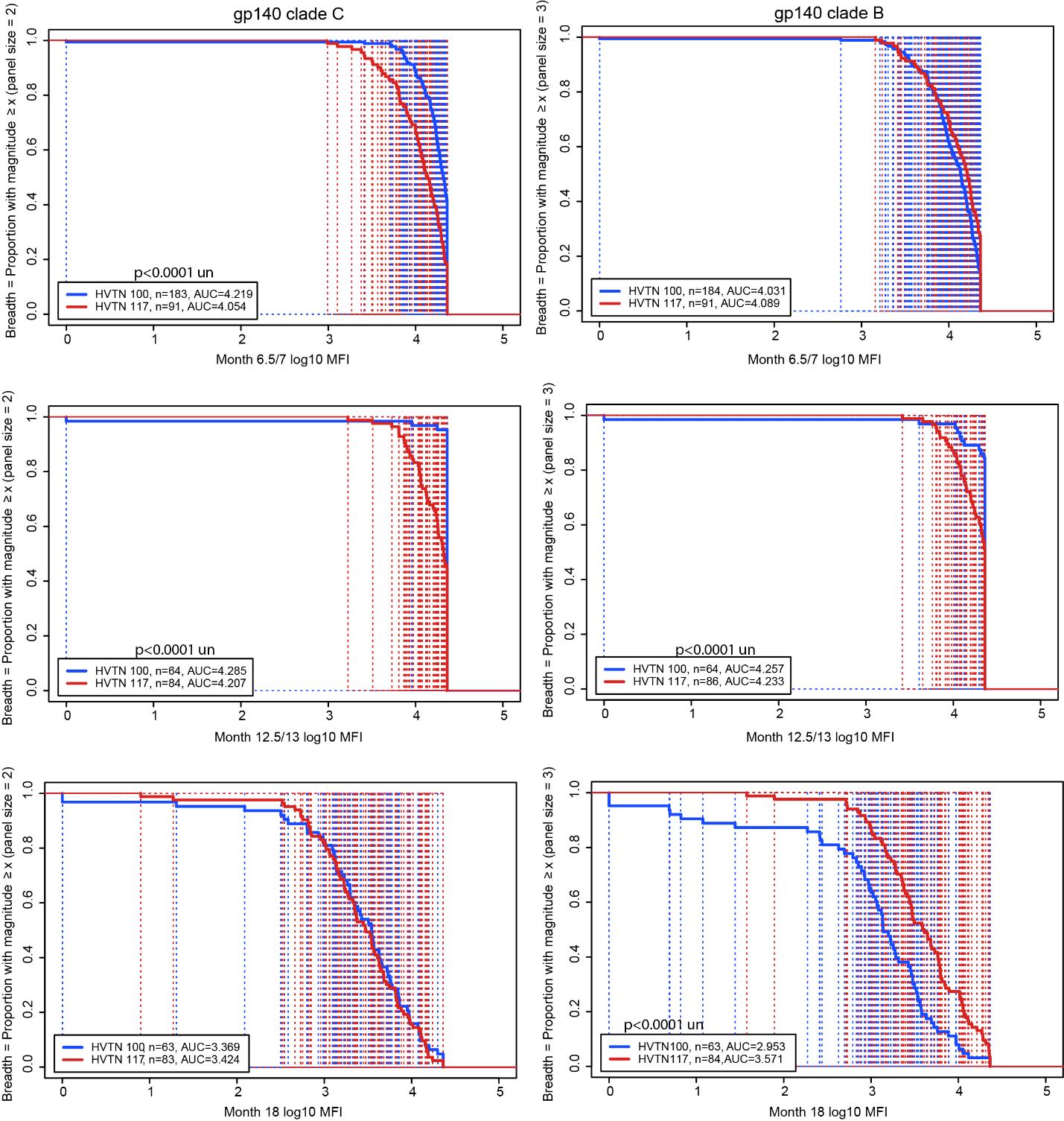


**Fig B.** **Magnitude-breadth of IgG gp140 Responses.**

Magnitude-breadth curves *summarizing IgG responses to the gp140 clade C (left) and gp140 clade B (right) antigen panels at month 6.5/7 (top row), month 12.5/13 (middle), and month 18 (bottom row). Unadjusted AUCs are reported in the bottom left corner of each panel. Un = unadjusted p value.*


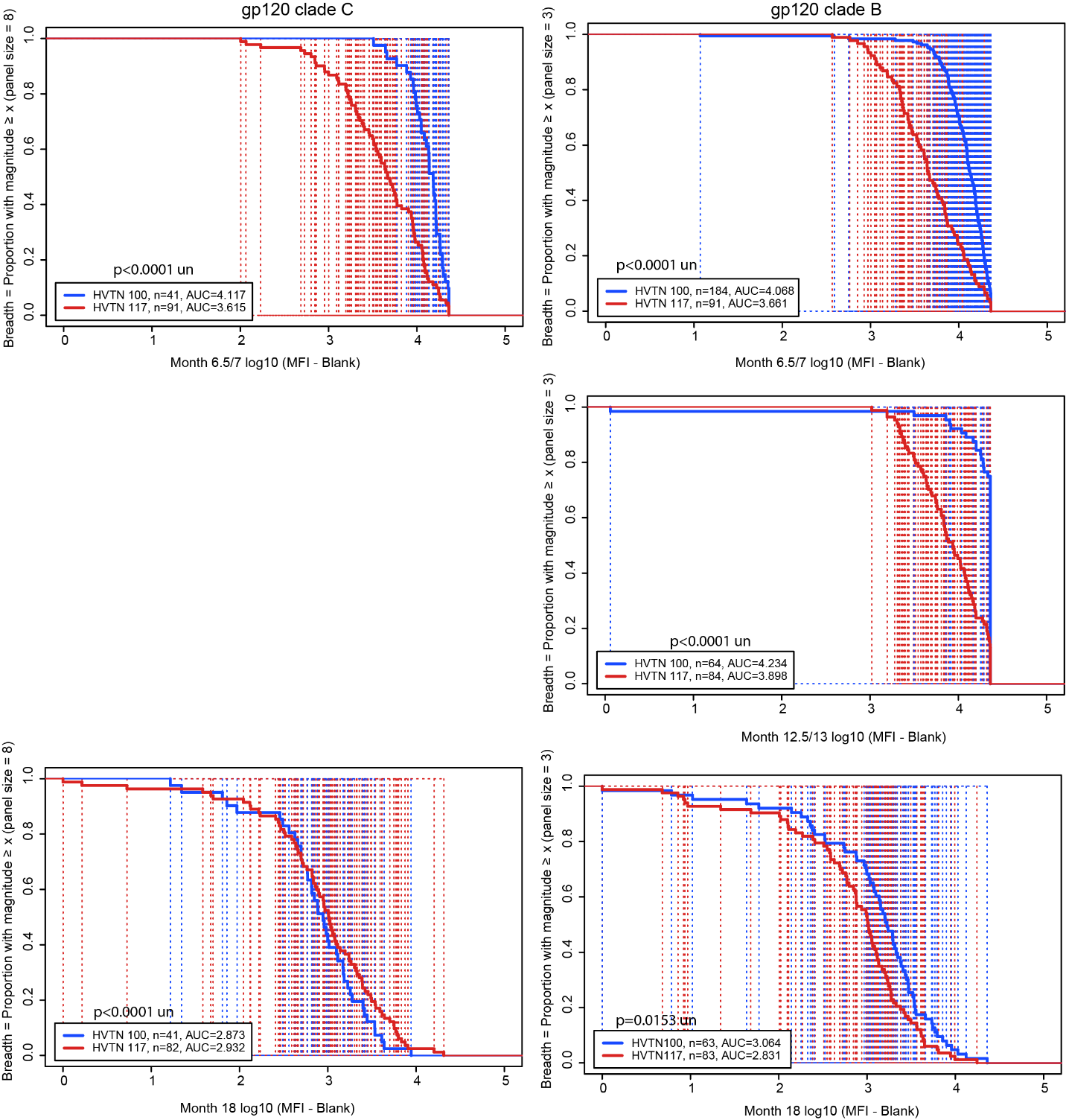


**Fig C.** **Magnitude-breadth of IgG gp120 Responses.**

Magnitude-breadth curves summarizing IgG responses to the gp120 clade C (right) and gp120 clade B (left) antigen panels at month 6.5/7 (top row), month 12.5/13 (middle), and month 18 *(bottom row). Unadjusted AUCs are reported in the bottom left corner of each panel. Un = unadjusted p value.*


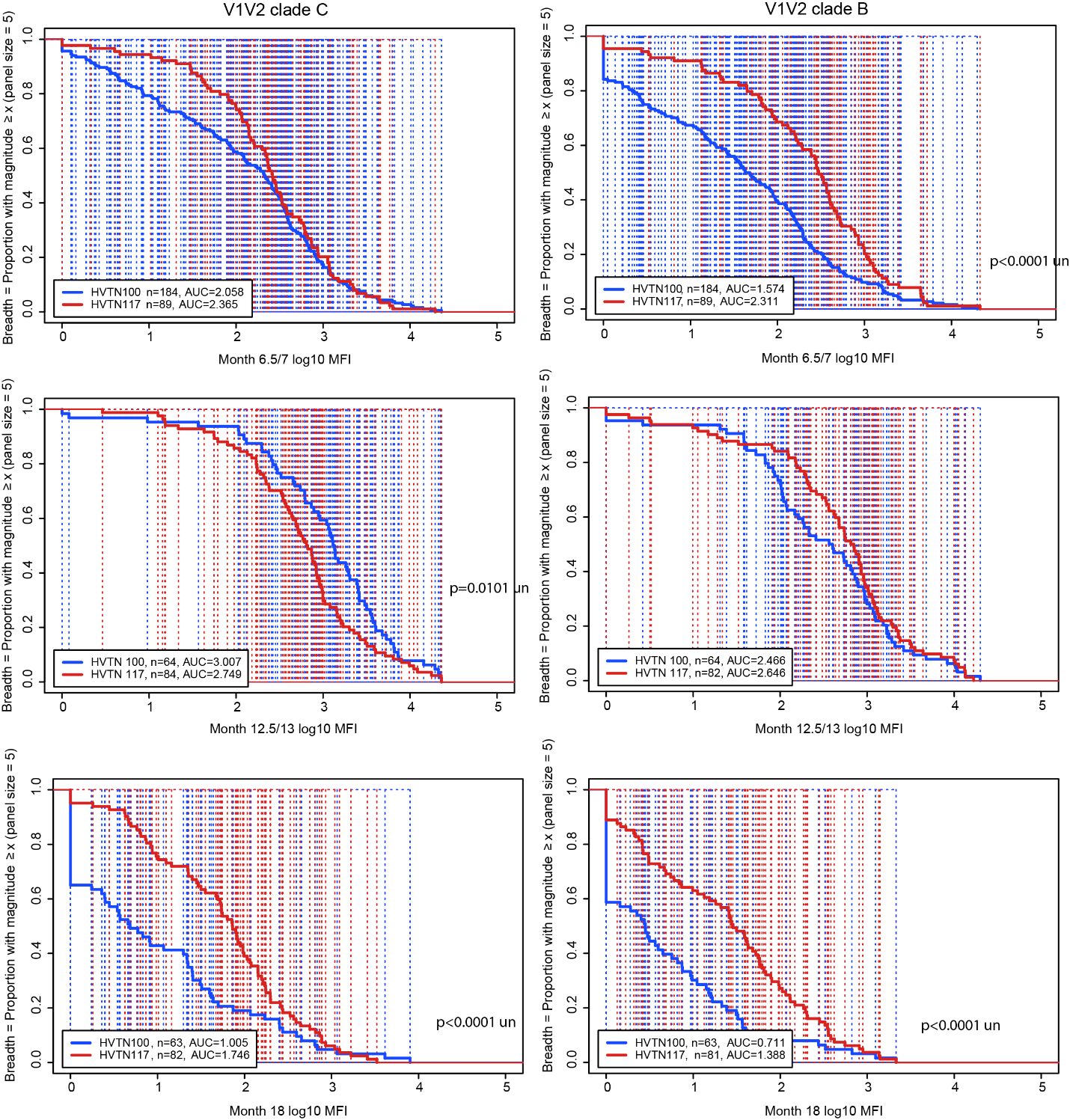


**Fig D. Magnitude-breadth of V1V2 IgG Responses.**

Magnitude-breadth curves summarizing IgG responses to the V1V2 clade C (/left) and V1V2 clade B (right) antigen panels at month 6.5/7 (top row), month 12.5/13 (middle), and month 18 (bottom row). *Unadjusted AUCs are reported in the bottom left corner of each panel. Un = unadjusted p value.*

**
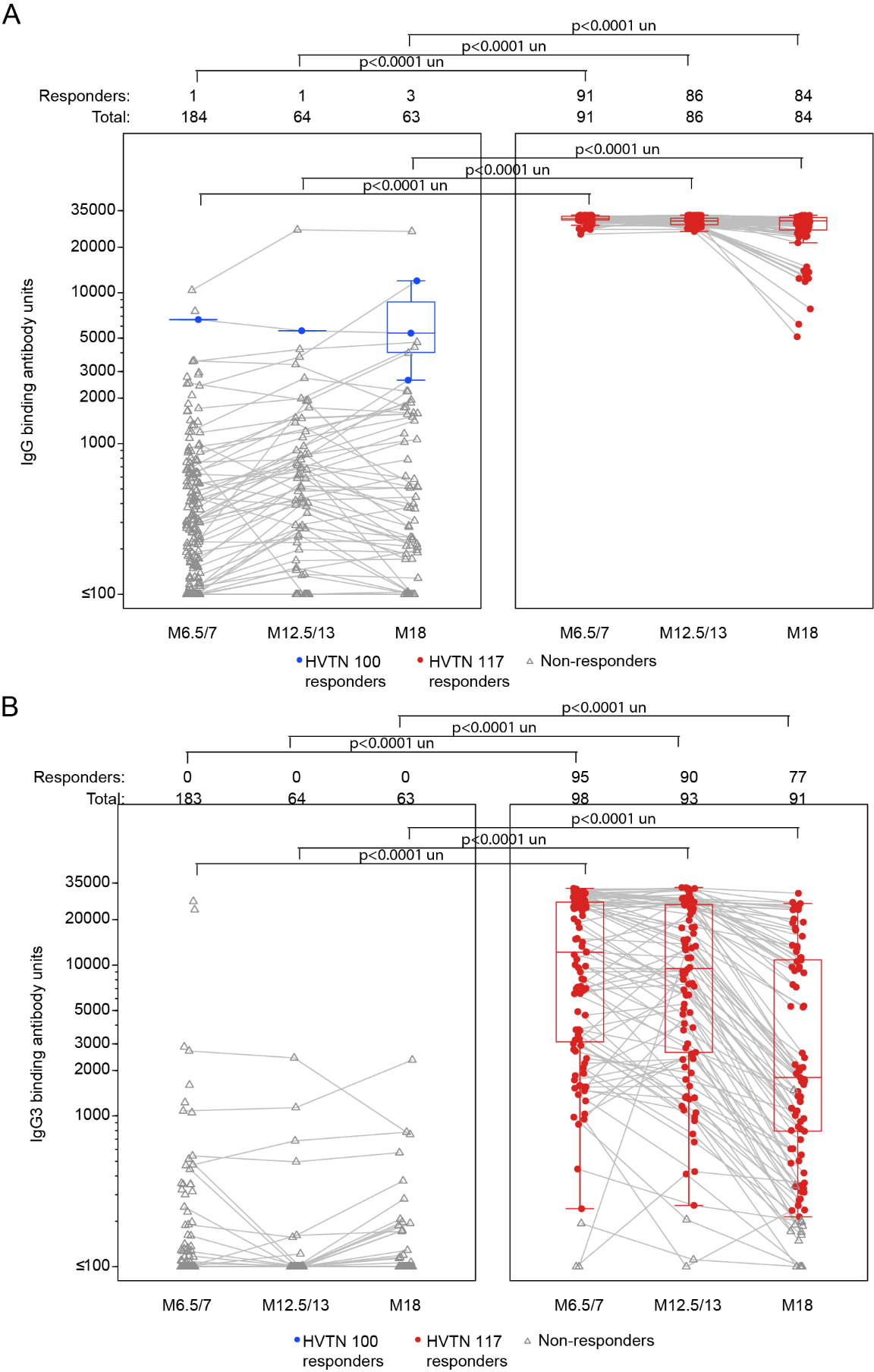
**

**Fig E. Summary of IgG (A) and IgG3 (B) responses to gp41.**

Boxplots summarize the magnitude of IgG3 responses among positive responders (shown as colored circles), *negative responders are shown as gray triangles. Gray lines connect observations from the same study participant. Un = unadjusted p value.*

**Fig F. Summary of CD4+ T-cell vaccine-matched Gag peptides to (A) IL-2 and/or IFN-γ and (B) polyfunctionality scores over time, and (C) magnitudes of marker-specific CD4+ T-cell responses at Month 12.5/13.**


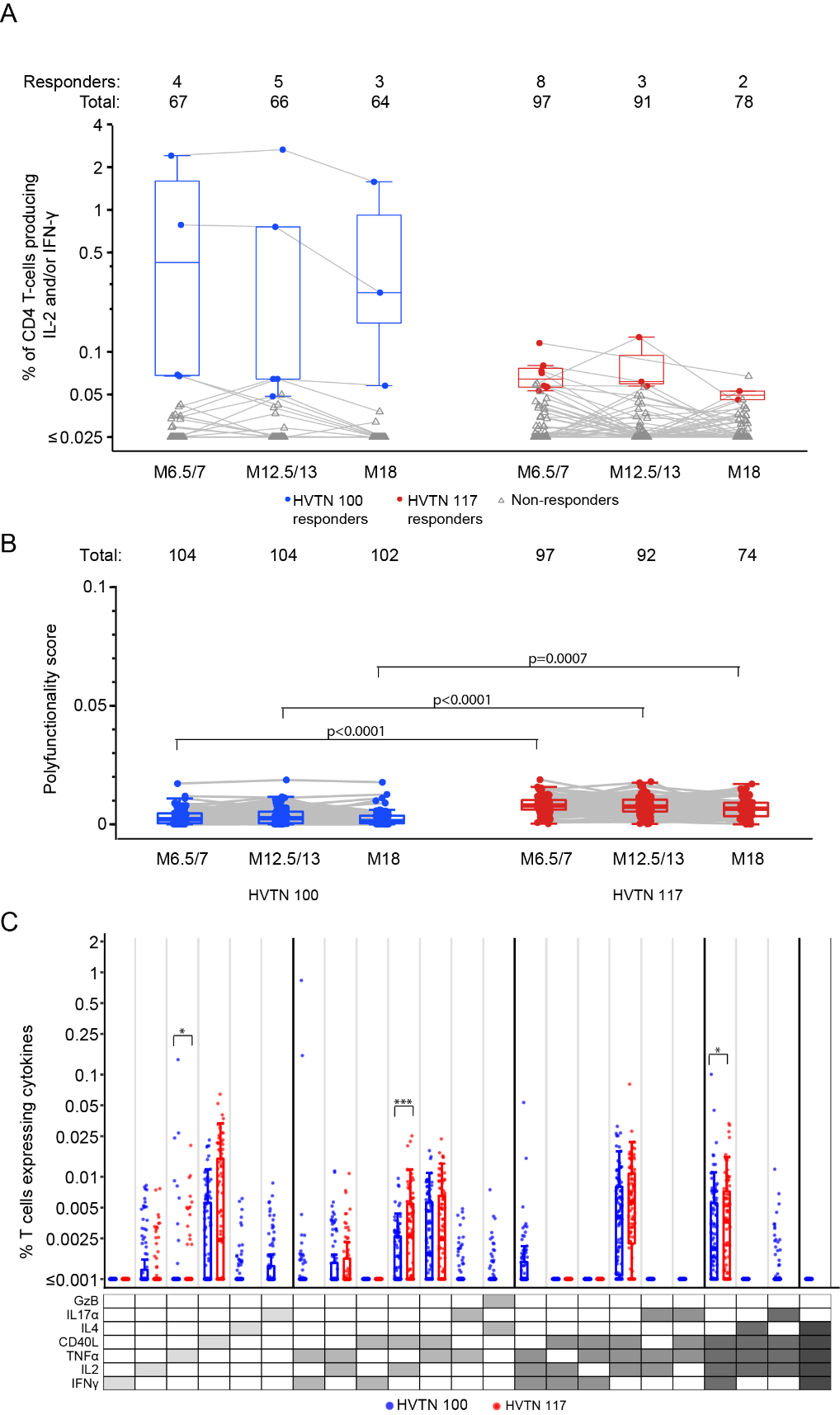


Boxplots showing IL-2 and/or IFN-γ responses and are based on positive responders only (shown as colored circles), negative responders are shown as gray triangles. Gray lines connect observations from the same study participant. Along the top panels A and B, Total represents the number of study participants analyzed at each time point, and Responders the number of those who had a positive response. Boxplots showing the CD4+ T-cell polyfunctionality scores, stratified by protocol (left). Lines connect scores for the same participant. Boxplots summarize marker-specific CD4+ T-cell responses at Month 12.5/13 (right), ordered according to the marker legend in gray where marker subsets are denoted by the markers they express (white, “off”; shaded “on”) and ordered from single function to higher-degree functionality. Cytokine combinations with COMPASS-estimated average posterior probabilities of at least 0.005 for either protocol are shown. TMLE p values are shown. In part C, * is p<0.05; and *** is p<0.001*.*

**Fig G. Summary of CD8+ T-cell vaccine-matched Gag peptides to (A) IL-2 and/or IFN-γ and (B) polyfunctionality scores over time, and (C) magnitudes of marker-specific CD8+ T-cell responses at Month 12.5/13.**


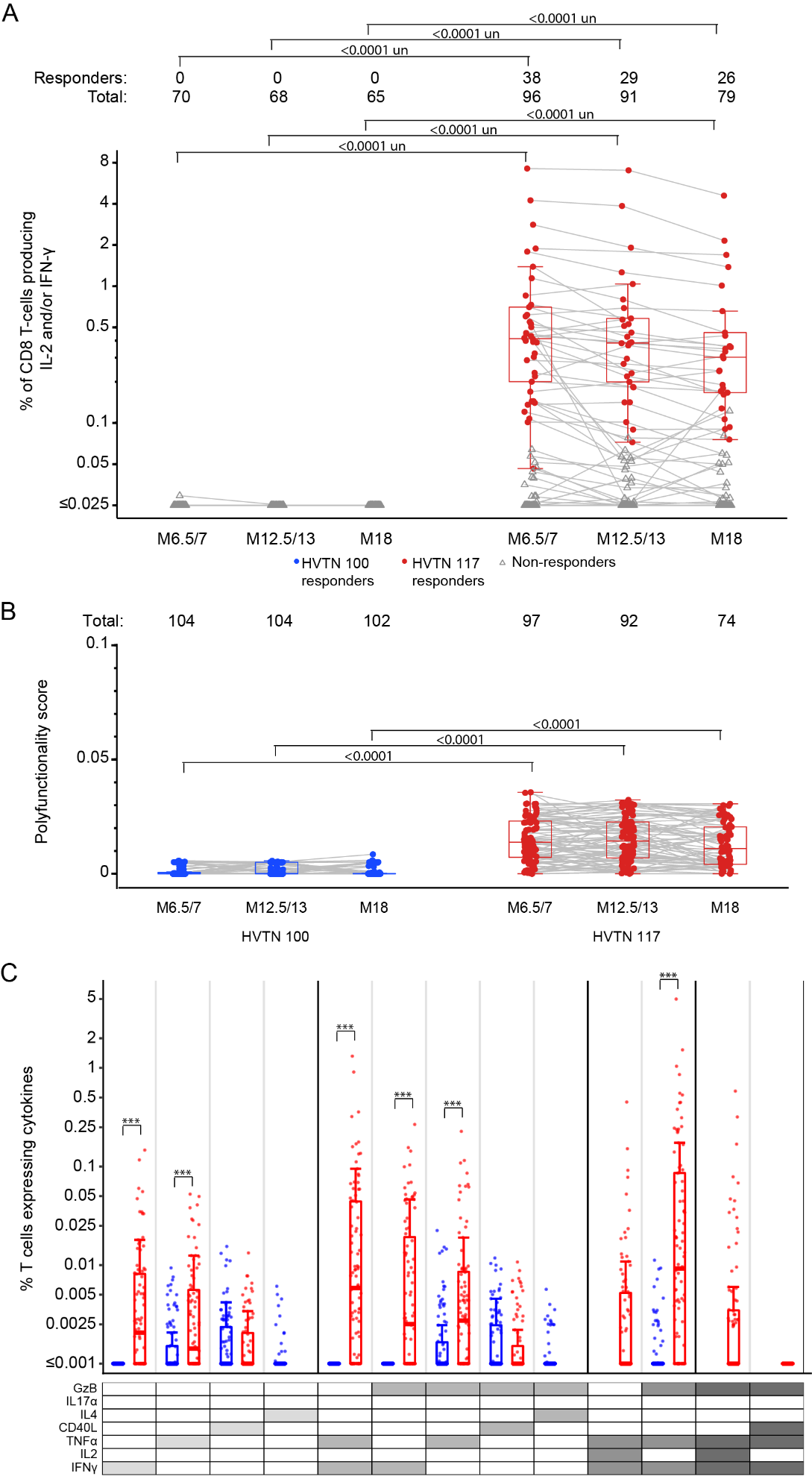


Boxplots showing IL-2 and/or IFN-γ responses and are based on positive responders only (shown as colored circles), negative responders are shown as gray triangles. Gray lines connect observations from the same study participant. Along the top panels A and B, Total represents the number of study participants analyzed at each time point, and Responders the number of those who had a positive response. Boxplots showing the CD8+ T-cell polyfunctionality scores, stratified by protocol (left). Lines connect scores for the same participant. Boxplots summarize marker-specific CD8+ T-cell responses at Month 12.5/13 (right), ordered according to the marker legend in gray where marker subsets are denoted by the markers they express (white, “off”; shaded “on”) and ordered from single function to higher-degree functionality. Cytokine combinations with COMPASS-estimated average posterior probabilities of at least 0.005 for either protocol are shown. *Un=unadjusted p value.* *In part C, * is *** is p<0.001.*

**REFERENCES**

1. Huang Y, Gilbert PB, Montefiori DC, Self SG. Simultaneous Evaluation of the Magnitude and Breadth of a Left and Right Censored Multivariate Response, with Application to HIV Vaccine Development. Stat Biopharm Res. 2009;1(1):81-91. Epub 2010/01/15. doi: 10.1198/sbr.2009.0008. PubMed PMID: 20072667; PubMed Central PMCID: PMCPMC2805400.

2. Horton H, Thomas EP, Stucky JA, Frank I, Moodie Z, Huang Y, et al. Optimization and validation of an 8-color intracellular cytokine staining (ICS) assay to quantify antigen-specific T cells induced by vaccination. J Immunol Methods. 2007;323(1):39-54. Epub 2007/04/25. doi: 10.1016/j.jim.2007.03.002. PubMed PMID: 17451739; PubMed Central PMCID: PMCPMC2683732.

3. De Rosa SC. Vaccine applications of flow cytometry. Methods. 2012;57(3):383-91. Epub 2012/01/19. doi: 10.1016/j.ymeth.2012.01.001. PubMed PMID: 22251671; PubMed Central PMCID: PMCPMC3349786.

4. Benkeser D, Carone M, Laan MJV, Gilbert PB. Doubly robust nonparametric inference on the average treatment effect. Biometrika. 2017;104(4):863-80. Epub 2018/02/13. doi: 10.1093/biomet/asx053. PubMed PMID: 29430041; PubMed Central PMCID: PMCPMC5793673.
